# Efficacy of a Spatial Repellent for Control of Malaria in Indonesia: A Cluster-Randomized Controlled Trial

**DOI:** 10.4269/ajtmh.19-0554

**Published:** 2020-05-18

**Authors:** Din Syafruddin, Puji B. S. Asih, Ismail Ekoprayitno Rozi, Dendi Hadi Permana, Anggi Puspa Nur Hidayati, Lepa Syahrani, Siti Zubaidah, Dian Sidik, Michael J. Bangs, Claus Bøgh, Fang Liu, Evercita C. Eugenio, Jared Hendrickson, Timothy Burton, J. Kevin Baird, Frank Collins, John P. Grieco, Neil F. Lobo, Nicole L. Achee

**Affiliations:** 1 Eijkman Institute for Molecular Biology, Jakarta, Indonesia;; 2 Department of Parasitology, Faculty of Medicine, Universitas Hasanuddin, Makassar, Indonesia;; 3 Department of Epidemiology, Faculty of Public Health, Universitas Hasanuddin, Makassar, Indonesia;; 4 Public Health and Malaria Control, PT Freeport Indonesia, International SOS, Kuala Kencana, Papua, Indonesia;; 5 The Sumba Foundation, Public Health and Malaria Control, Bali, Indonesia;; 6 Department of Applied and Computational Mathematics and Statistics, University of Notre Dame, Notre Dame, Indiana;; 7 Center for Computer Research, University of Notre Dame, Notre Dame, Indiana;; 8 Department of Biological Sciences, Eck Institute for Global Health, University of Notre Dame, Notre Dame, Indiana;; 9 Eijkman-Oxford Clinical Research Unit, Jakarta, Indonesia;; 10 Nuffield Department of Medicine, Centre for Tropical Medicine, University of Oxford, Oxford, United Kingdom

## Abstract

A cluster-randomized, double-blinded, placebo-controlled trial was conducted to estimate the protective efficacy (PE) of a spatial repellent (SR) against malaria infection in Sumba, Indonesia. Following radical cure in 1,341 children aged ≥ 6 months to ≤ 5 years in 24 clusters, households were given transfluthrin or placebo passive emanators (devices designed to release vaporized chemical). Monthly blood screening and biweekly human-landing mosquito catches were performed during a 10-month baseline (June 2015–March 2016) and a 24-month intervention period (April 2016–April 2018). Screening detected 164 first-time infections and an accumulative total of 459 infections in 667 subjects in placebo-control households, and 134 first-time and 253 accumulative total infections among 665 subjects in active intervention households. The 24-cluster protective effect of 27.7% and 31.3%, for time to first-event and overall (total new) infections, respectively, was not statistically significant. Purportedly, this was due in part to zero to low incidence in some clusters, undermining the ability to detect a protective effect. Subgroup analysis of 19 clusters where at least one infection occurred during baseline showed 33.3% (*P*-value = 0.083) and 40.9% (*P*-value = 0.0236, statistically significant at the one-sided 5% significance level) protective effect to first infection and overall infections, respectively. Among 12 moderate- to high-risk clusters, a statistically significant decrease in infection by intervention was detected (60% PE). Primary entomological analysis of impact was inconclusive. Although this study suggests SRs prevent malaria, additional evidence is required to demonstrate the product class provides an operationally feasible and effective means of reducing malaria transmission.

## INTRODUCTION

It has been nearly 75 years since the role of spatial repellency (deterrence or avoidance) was first described as a potentially beneficial attribute in malaria control, showing chemicals could effectively disrupt normal host-seeking mosquito behavior and interrupt contact with humans, thus preventing disease transmission.^1–3^ Spatial repellency is used here as a general term to refer to a range of insect behaviors induced by airborne, volatile chemicals that ultimately result in a reduction in human–vector contact. These behaviors include movement away from a treated space with chemical stimulus, interference with host detection (attraction-inhibition), and/or interference with feeding response (feeding-inhibition).^4–7^ Spatial repellency can be measured and distinguished from other chemical actions, primarily contact irritancy and toxicity, in the laboratory^8,9^ and in semi-field evaluations.^10,11^ Which behavior is elicited by a volatile chemical depends on the concentration or dose–exposure interaction by the mosquito in the treated space. For example, toxicity occurs at higher chemical doses, whereas deterrence (behavioral avoidance) can result from lower, sublethal chemical concentrations.^12^ Currently, most commercial spatial repellent (SR) products use either low concentrations of short-duration United States Environmental Protection Agency (USEPA) registered synthetic pyrethroids (pyrethrin, metofluthrin, and more recently transfluthrin)^13^ or botanical-based compounds.^14,15^

The WHO Vector Control Advisory Group (VCAG) assesses evidence on the epidemiological effectiveness of new vector control interventions and by doing so supports WHO’s development of policy recommendations, including the potential use of SRs as a public health vector control strategy.^16^ Reviewed by VCAG since 2014, assessment of rigorous epidemiological evidence for endorsing a policy recommendation of a SR intervention class remains limited and deemed insufficient.^17^ A malaria prevalence study in China evaluating mosquito coils containing 0% transfluthrin demonstrated a 77% reduction in human *Plasmodium falciparum* cases,^18^ and the use of coils containing 0.00975% metofluthrin provided 52% protective efficacy (PE) against new (incident) malaria infections in Indonesia.^19^ Although findings from both studies were encouraging, neither met the required VCAG evidence for full public health assessment because of lack of adequate scale in study design (small cluster number in Indonesia) and/or being underpowered (both studies).^20^ The importance of a WHO policy for implementation of SRs in malaria control programs could dramatically increase investments by the private industry to develop chemicals that operate through modes of actions to elicit vector behavior changes other than purely toxicity. This would potentially introduce a new generation of effective active ingredients and product formulations into the disease control/eradication arsenal.^21,22^ In combination with existing WHO-recommended malaria control interventions, SRs may add protective benefit in reducing vector-borne diseases.^23^ This is most highlighted in settings where traditional long-lasting insecticidal nets (LLINs) or indoor residual spraying (IRS) may not be sufficiently protective because of varying circumstances: 1) early-evening blood-feeding vectors,^24,25^ 2) when LLINs are not in use or used intermittingly,^26,27^ 3) where vectors do not or limit resting time indoors on insecticide-treated surfaces,^24,25^ and 4) when LLINs and/or IRS are unavailable, or are impractical and/or infeasible such as during humanitarian emergency relief operations.^28^ Control or elimination of malaria in these circumstances will require innovative approaches, for example, SRs providing a highly beneficial protective role against transmission.^29,30^

The current claim of an SR intervention class for public health value is “deployment of a spatial repellent will prevent human–vector contact to reduce pathogen transmission.” The role of spatial repellency in public health is dependent on assessing whether or not human protection is rigorously evidenced, thus the requirement of clinical trials using a prototype SR product for the intervention class. With this epidemiological primary end point in mind, the objectives of the current randomized cluster trial (RCT) were to build on previous epidemiological findings and provide rigorous evidence of an SR that provides a sufficient protective effect against malaria infection risk and demonstrate risk reduction by decreasing relevant entomological measures (e.g., anopheline human-landing (biting) density, age-grading parity, and/or sporozoite infectivity rates) in endemic communities. The rationale for conducting the RCT on Sumba Island stems from an essential requirement by the WHO VCAG that more evidence of human health impact is needed to recommend a WHO global public health SR policy^31^ and that such evidence should come from varied malaria endemicity settings (low, moderate, and high).^16^ Sumba Island was selected as the study site based on the following criteria: 1) active malaria transmission, 2) vector populations exhibiting early-evening and/or outdoor biting patterns, and 3) housing characteristics which reflect probability of indoor exposure to mosquitoes. The underlying reason for integrating entomological measures (aforementioned) in the RCT was to identify correlations, if any, with PE, which could then be used in future trials with appropriate minimum thresholds required to demonstrate non-inferiority of next-generation SR products.

## METHODS

The study was conducted from June 2015 to April 2018, registered in clinicaltrials.gov (Identifier: NCT02294188), and performed according to the International Conference on Harmonization’s Guideline for Good Clinical Practice (GCP; document E6) (R1) and the Organization for Economic Co-operation and Development (OECD) Principles of Good Laboratory Practice based on the National Agency of Drug and Food Control, Indonesia regulations.^32,33^ There were no changes to the methods after trial commencement.

### Ethics statement.

Ethical review and approval for this study was granted by the Ethics Committee (EC) of the Faculty of Medicine, Universitas Hasanuddin (Protocol #UH14070385) and the University of Notre Dame (Protocol #14-01-1448) and endorsed by the Eijkman Institute Research EC, Jakarta, Indonesia. Consent was obtained from parents or guardians of child recruits following EC guidelines. For sentinel households participating in entomological collections, signed consent was sought from the head of household. No data on individuals were collected during the entomological collections. During the consenting process, the study was described and the relevant consent form read in local dialect by the study staff. The consent form detailed the design of the study, including radical cure, the purpose of collecting and storage of blood samples (toward laboratory-based malaria diagnosis), descriptions of the study risks, benefits, and procedures of therapeutic radical cure and follow-up. For households recruited for entomology measures, the consent form detailed the design of mosquito collections and descriptions of study risks, benefits, and procedures of human-landing catch (HLC).

Participants who were illiterate were asked to appose their thumbprint and, when possible, a literate witness was asked to sign (i.e., a witness selected/known by the participant and having no connection to the research team). All households were provided with a signed copy of the consent form after agreeing to participate in the study. Adverse events (AEs) were captured during participant follow-up and entomological collections and reported to monitoring authorities in accordance with the approved protocol. All participants were free to drop out of the study at any time, regardless of the reason or having to provide one, and without prejudice.

### Study setting.

**Figure 1.**
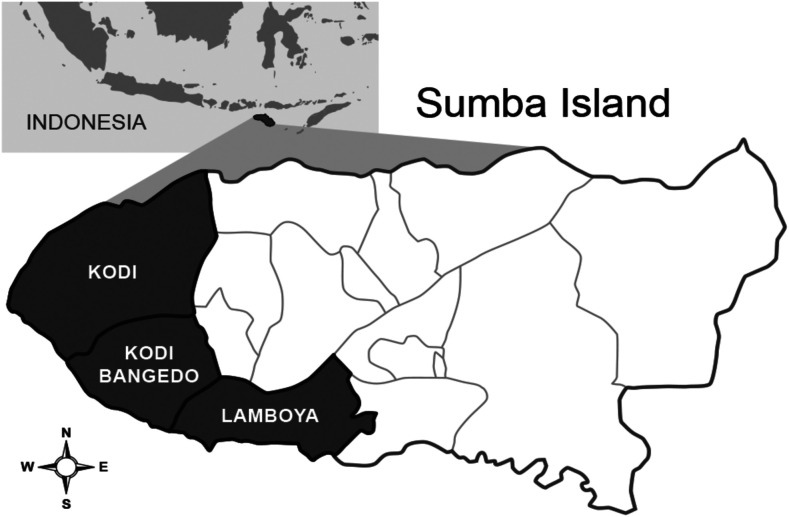
Study site areas in Southwest and West Sumba districts located in Kodi, Kodi Bangedo, and Lamboya subdistricts of Sumba Island, Nusa Tenggara Timur Province (eastern Lesser Sunda Islands), Indonesia (map not to scale).

The study was conducted in Southwest and West Sumba districts, East Nusa Tenggara Province, Indonesia (Figure 1). The circa 400,000 residents of the two districts occupy 175 village “groups” (*desas*) and several small-sized towns.^34^ Thirteen village groups, with resident populations ranging between 1,067 and 3,904 (avg. 2,132), served as study locations for the final selection of the 24 study clusters consisting of multiple desas. Organized bed net campaigns were recently introduced with the last mass distribution occurring in February 2018–March 2018 across the study area. A mass distribution of LLINs occurred in October–December 2014 in both districts (Olyset Net, permethrin 2.0% w/w) with a reported > 95% coverage of households provided 1–4 nets each. A second round of LLIN mass distribution occurred in February 2018–March 2018 (PermaNet^®^ 3.0, Vestergaard Frandsen SA, Denmark, deltamethrin 180 mg/m^2^ + piperonyl butoxide synergist). The last round of focal IRS occurred in 2003 using a pyrethroid-based product. The malaria prevalence based on microscopically diagnosed parasitemia drawn from a random sample of 50% of residents present in 13 villages conducted in 2015, 10 months before the intervention trial, averaged 15.5% (2.5–37.3%) (Table 1).

**Table 1:** Malaria infection prevalence data from 10 months before randomization in the 13 villages from which study clusters were delineated on Sumba Island

Village	Number of samples	Number of malaria infections	Slide positive (%)
Matakapore	806	110	13.65
Manutoghi	622	217	34.88
Waimakaha	621	149	23.99
Wailangira	857	52	6.07
Waikarara	1,420	63	4.44
Panenggo Ede	610	15	2.46
Waimaringi	649	67	10.32
Tana Mete	810	81	10.00
Kahale	903	202	22.37
Rada Malando	402	150	37.31
Karang Indah	523	195	37.28
Gaura	1,297	165	12.70
Weetana	1,129	163	14.40

**Figure 2.**
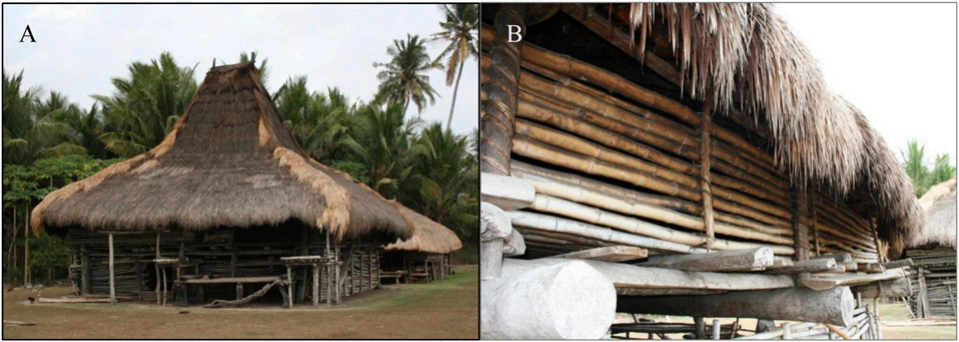
Traditional Sumba house structure (**A**) raised ∼1 m aboveground and averaging ∼70 m^3^ in size with thatch roof, bamboo floors, and walls (**B**), which offer minimal protection from mosquito entry.

Although very little detail is known about the malaria vector distribution and bionomics in this study area,^35^ an earlier (August 2007) entomologic survey documented 13 species of anophelines occurring in West Sumba district: *Anopheles aconitus* (Dönitz), *Anopheles annularis* Van der Wulp, *Anopheles barbirostris* s.l. (Satoto), *Anopheles flavirostris* (Ludlow), *Anopheles hyrcanus* group (Reid), *Anopheles indefinitus* (Ludlow), *Anopheles kochi* (Dönitz), *Anopheles leucosphyrus* group (Reid), *Anopheles maculatus* (Theobald), *Anopheles subpictus* s.l. (Grassi), *Anopheles sundaicus* s.l. (Dusfour et al.), *Anopheles tessellatus* (Theobald), and *Anopheles vagus* (Dönitz).^36^ These species vary spatially in relative abundance associated with the presence of their preferred aquatic larval habitats ranging from coastal brackish and freshwater marshes and ponds, seasonally productive rice paddies, to forested hillsides with perennial running streams and small rivers. At the time of the survey, human-landing collections revealed *An. subpictus* and *An. vagus* as the predominant species in the upland interior locations and *An. sundaicus* as the most common species attracted to humans along the coastal plain. Most residents in the study villages work as small-holder agriculturalist pursuits, typically lacking public supplied electricity or articulated water supply systems. Homes are predominately traditional designed, large, thatched-roof structures raised ∼1 m aboveground, averaging ∼70 m^3^ in size (6 m length × 6 m width × 2 m in height) and constructed with gaping bamboo walls and flooring that offer little protection from mosquito entry (Figure 2).

### Trial design.

**Figure 3.**
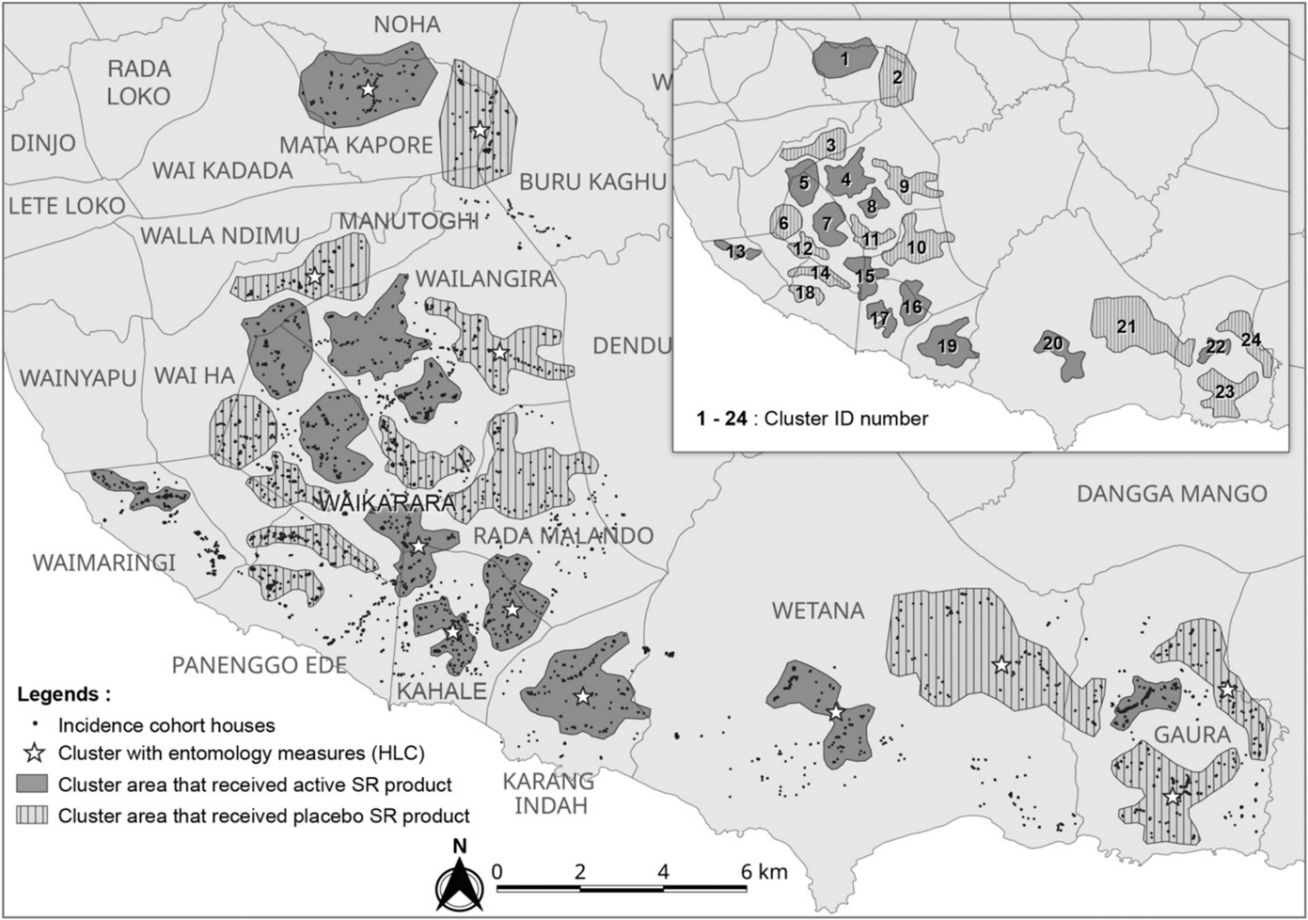
Location of 24 study clusters in West and Southwest districts, Sumba. Clusters were selected for enrolling the incidence cohort, each consisting of ca. 100 households with an average distance of 500 m between clusters. A total of 48 sentinel houses from 12 clusters were selected for routine entomological human-landing catch.

The study was a randomized cluster, double-blind, placebo-controlled, clinical trial with a total of 24 clusters divided into 12 clusters per intervention arm. Clusters were selected based on housing type, focusing on traditional houses (with or without thatch roofing) (Figure 2), mitigation of potential chemical dispersion “spillover” effect (i.e., distance between nearest homes in different clusters ∼500 m apart), and logistical considerations (i.e., distance from field-based satellite laboratories). About 54 households were recruited within each cluster based on human sample size requirements. Households from 13 villages were stratified into 24 clusters before randomization: Gaura (pop. 2,831; houses 432), Kahale (pop. 3,904; houses 253), Karang Indah (pop. 1,169; houses 139), Manutoghi (pop. 1,067; houses 165), Matakapore (pop. 2,805; houses 209), Panenggo Ede (pop. 1,271; houses 90), Rada Malando (pop. 1,842; houses 133), Tana Mete (pop. 2,124; houses 213), Waikarara (pop. 2,709; houses 446), Wailangira (pop. 1,878; houses 226), Waimakaha (pop. 2,334; houses 211), Waimaringi (pop. 2,598; houses 116), and Weetana (pop. 2,670; houses 275) (Figure 3).

### Sample size.

Previous malaria incidence rates collected in a portion of the study area in coastal Kodi subdistrict were used to estimate the likely malaria attack rate in the current study villages at 0.3 infections/person-year.^19^ Sample size determination was based on the hazard rate comparison in the proportional hazards regression model.^37^ The required number of first-time infections was estimated at 417 to permit detection of a 30% protective effect by the SR intervention compared with placebo with 80% power at the type-I error rate, assuming a between-cluster coefficient of variation (CV; defined as the ratio between SD and mean) of 30% and a baseline hazard rate of 0.3 per person-year. With 12 clusters per treatment arm (active or placebo), with 2-month accrual and a 22-month follow-up, and an estimated 20% loss to follow-up (LTFU) during intervention, a total of 54 subjects per cluster were required (*n* = 1,296). As entomology measures were used in relationship analyses of PE rather than hypothesis testing, the sample size was based on field logistical capacity and not statistical power.

### Participants.

The average number of children ≤ 5 years of age in each cluster was 68 (57–79). From individual households in study clusters, one child, aged ≥ 6 months to ≤ 5 years at the time of recruitment, was provided the opportunity to enroll as a subject in the study. Following informed consent, medical screening consisted of physical examination by a study physician and a qualitative nicotinamide adenine dinucleotide phosphate (NADPH) spot test for G6PD deficiency (Trinity Biotech qualitative glucose-6-phosphate dehydrogenase (G6PD) assay, ref 345-UV, Trinity Biotech, St. Louis, MO).^38^ In addition to being of G6PD normal status, eligibility requirements included bodyweight ≥ 40 kg, hemoglobin > 5 mg/dL (Hb201+, HemoCue AB, Angelholm, Sweden),^39^ no severe acute illness/infection on the day of inclusion, temperature ≤ 38°C, participant acknowledging sleeping in the village > 90% of nights during any given month, not participating in another clinical trial, and no intensions for extended travel during the study period.

### Intervention.

A 24-month intervention follow-up period was implemented from April 2016 to April 2018. Intervention was simultaneously initiated in all subject households and nonsubject households that consented to receive intervention. The SR intervention is a transfluthrin-based passive emanator produced by S.C. Johnson & Son, Inc. (SCJ) designed to release a volatile chemical into the air without the requirement of an external heat source, such as electricity or fire, and prevent human–vector contact by disrupting normal behavior of mosquitoes at a distance within the treated space. Indoor placement of the intervention product was designed with the objective to measure PE under indoor use conditions. Transfluthrin is a registered compound commonly found in commercially available mosquito coils globally based on WHO specifications.^40^ The USEPA has recently approved transfluthrin products for indoor use within the United States.^41^ Emanators (active and placebo) of identical packaging and color were distributed by study personnel every 2 weeks at the individual household application rate of 2 units/9 m^2^ according to SCJ specifications. There was a median of one room (range 1–9) and 10 emanators (range 4–56) per household. Intervention devices were positioned indoors by hanging individual emanators on two metal hooks specially attached to walls for this study. Each position remained static throughout the study. The hooks facilitated stabilization of the interventions, so the chemical-treated surface was consistently exposed facing the interior space. Research staff placed, removed, and replaced emanators in households at set intervals and recorded attrition, based on the number of products removed from each structure during each replacement period, to ascertain the application rate for use in estimating cluster coverage. During the trial period, quality control analysis was performed by Ross Laboratories, India, on unused (in storage) emanators to verify the amount of transfluthrin in actives and the absence of transfluthrin in placebos. At the end of the follow-up period (April 2018), used, unused, and expired emanators were disposed of by PT. Wahana Pamunah Limbah Industri in Jakarta according to Indonesian regulations on disposal requirements.

### Randomization, allocation, and blinding.

Clusters were allocated to receive either active or placebo treatment using a random number generator (https://www.random.org). The cluster allocation code was made available from the intervention manufacturer to the Data Safety Monitoring Board (DSMB) for use in safety assessments. The site database manager assigned a unique household identification number (HIN) to each structure, and the site intervention administrator coordinated the distribution of the blinded active or placebo to enrolled households within each cluster corresponding to the pre-labeled package code. Unblinded assignments were shared with a site administrator in a sealed envelope placed in a secure location within the managing center of the research project (Jakarta) for purposes of emergency unblinding related to AE and serious adverse events (SAEs). Thus, the investigators, research team, study subjects, and residents were blinded as to which cluster received active versus placebo devices until after completion of the study.

### Procedures.

#### Radical cure and follow-up

**Figure 4.**
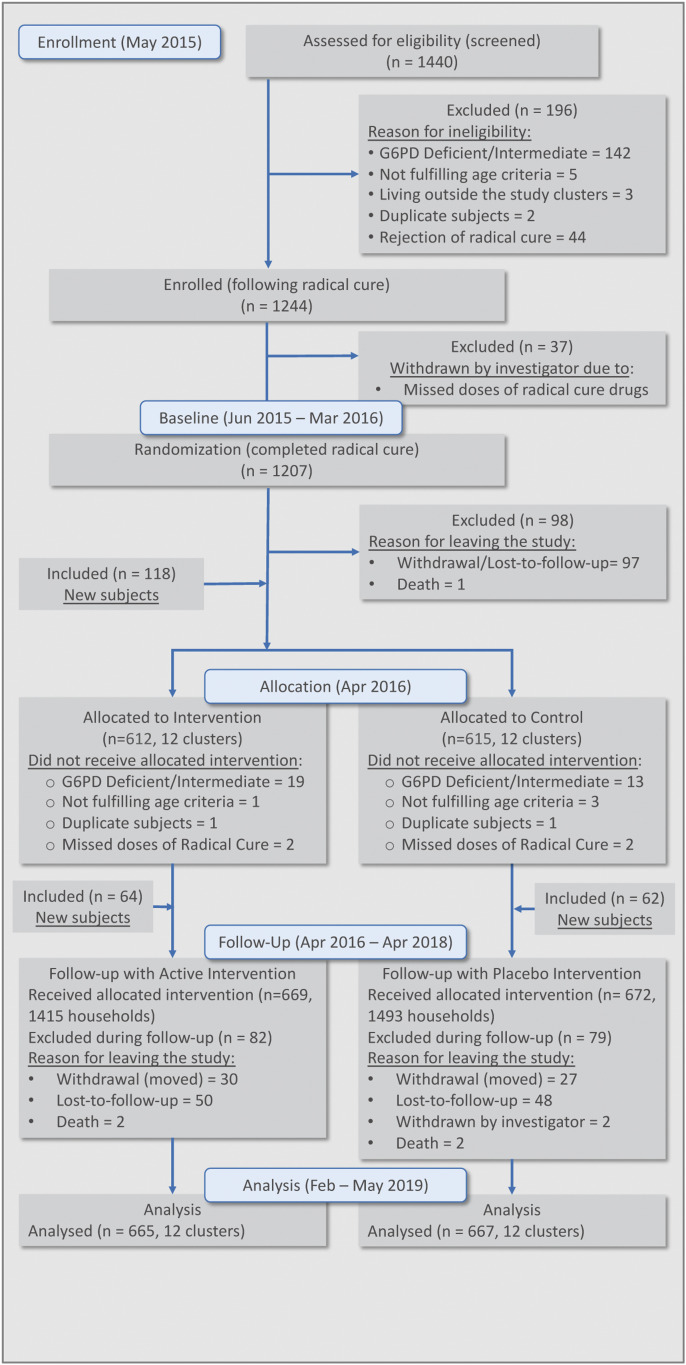
Flowchart of enrollment of study volunteers.

Figure 4 summarizes the screening, enrollment, and follow-up of the incidence density cohorts. The trial consisted of a 10-month baseline follow-up period (June 2015–March 2016) and a 24-month intervention follow-up period (April 2016–April 2018). A total of 1,353 subjects were presumptively radically treated using a fixed combination formulation of dihydroartemisinin–piperaquine (DHA-P) (containing 40 mg dihydroartemisinin and 320 mg piperaquine [Zhejiang Holley Nanhu, Beijing Holley Cotec]) administered as a weight per dose regimen of 2.25 and 18 mg/kg per dose of dihydroartemisinin and piperaquine, respectively, once daily for 3 days. Primaquine (PT, Pharos Tbk, Semarang, Indonesia) 0.25 mg/kg body weight was prescribed for the 14 days immediately before implementing the intervention. The DHA-P combination is currently the first-line antimalarial drug for uncomplicated malaria treatment in Indonesia.

All subjects were examined for *Plasmodium* spp. infection by expert microscopy and later confirmed using polymerase chain reaction (PCR) from matched filter paper blood spot samples.^42^ New malaria infections among the participants were monitored every 4 weeks using microscopic examination of Giemsa-stained blood films according to WHO guidelines based on a minimum of 200 high-magnification (×1000 oil-immersion) thick blood film fields.^43^ Two certified expert microscopists independently (blinded) examined slides on-site at project-dedicated field laboratories. Polymerase chain reaction detection of parasite DNA was conducted at the Eijkman Institute for Molecular Biology (EIMB) central laboratory in Jakarta for all four *Plasmodium* species.^42^ A blood sample was defined as malaria “positive” for inclusion in incidence analyses if it met the criterion of having two diagnostic outcomes indicating the presence of the parasite (e.g., 2 × microscopy, 1 × microscopy + 1 × PCR, or 2 × PCR). All positive and 10% randomly selected negative samples diagnosed at the EIMB were retested at the University of Notre Dame. Discordant microscopy and PCR results required reexamination of both initial findings. Participants found to be malaria infected at point-of-care were immediately treated with DHA-P and were removed from contributing further to person-time at risk of first-time infection; however, they remained in the study to monitor the overall (total new) incidence for malaria infections and the expected number of infection cases averted.

#### Entomologic surveys

In a subset of 12 clusters, adult mosquito diversity and densities were measured using HLCs every 2 weeks from the start of the baseline through the end of the follow-up period in a subset of 12 clusters. Clusters for entomological sampling were selected based on exhibiting highest HLC densities during baseline sampling, along with the existence of mosquito larval habitats. The 12 clusters were hierarchically stratified on criteria of baseline HLC and then blindly allocated to the treatment arm to ensure a balanced recruitment (six clusters in each treatment group). Four neighboring sentinel houses within each of the 12 clusters were randomly selected for mosquito collections (*n* = 48). Collections were conducted at sentinel houses for one night every 2 weeks from paired active/placebo clusters (e.g., three pairs on Monday night and three pairs on Wednesday night) during the intervention. Teams of two collectors were assigned per house, one positioned indoors near the center of the house and one located outside on the house verandah, approximately 1 m from the exterior wall. The collectors removed all mosquitoes landing on their exposed lower legs using a mouth aspirator. Collections were conducted from 18:00 to 06:00 hours for 50 minutes every hour. Paired collectors rotated between indoor and outdoor positions each hour. Samples were placed into individual holding containers labeled by collection hour, unique house code (linked to the blinded treatment code), and collection location (indoor or outside). Captured mosquitoes were immediately killed by ether-soaked cotton pads in the field and initially identified to species (or species complex) using morphological characters.^44^ All specimens were transported to an on-site base laboratory on completion of the 12-hour collection, and a random sample of representative anopheline species (up to 20% per cluster per indoor/outdoor location and hour of collection) was dissected for parity and scored as either gravid/parous or nulliparous.^45^ Partial (head-thorax for those dissected for parity) or whole anopheline specimens were placed singly into individual Eppendorf^®^ (Hamburg, Germany) 1.5-mL vials and stored over silica gel desiccant until further processing at the EIMB, Jakarta, for detection of malaria sporozoites and molecular-based species identification, where applicable.^46,47^ Mosquito samples were evaluated for *Plasmodium* species infection using PCR methodologies to derive corresponding malaria sporozoite infection rates by parasite and vector species.^48^ Together with time-adjusted HLC densities (anophelines/person-night), matched sporozoite rates were used to derive the entomological inoculation rates (EIRs) for each treatment arm.^49^ Anopheline species identification was verified at the University of Notre Dame following previous protocols (not reported herein).^46,47^

### Insecticide susceptibility assays.

Permethrin was evaluated using the WHO standard tube and CDC bottle assay during baseline, intervention, and post-intervention periods at the WHO-recommended discriminating concentration for anophelines of 0.8% and CDC-recommended dose of 21.5µg/mL active ingredient.^40,41^ Both WHO tube assays and CDC bottle assays were performed on F0 mixed anopheline species collected as immatures in 13 of the 24 study clusters from across 23 habitat locations during baseline, intervention, and 6-month post-intervention periods (ending October 2018). The assays were conducted using non–blood-fed, 3- to 5-day-old females according to established guidelines.^50,51^ After each test period, all chemical and control specimens were stored individually over silica gel for analysis at EIMB to confirm species identification and for detection of target-site mechanisms (e.g., *kdr* gene mutations) of resistance (not reported herein).

#### Monitoring of AEs and SAEs.

Adverse events, possibly related to transfluthrin exposure, in subjects and other household members, were captured by the study team during both active and passive blood sampling using a standardized survey form. Investigation of study-related AEs was performed by the on-site study clinician. Serious adverse events were also recorded according to the protocol, regardless of their possible relationship to the intervention. Government clinic health records were compiled on a quarterly basis starting in December 2017 for DSMB safety assessment of the study population.

#### Data management and verification

Data collection was designed around a tablet-based survey platform linked to a custom-built database and web portal. CommCare (Dimagi Inc, Cambridge, MA) was selected as the frontend form application, providing critical capabilities, including a parent–child case structure, the ability to store forms when offline, update form versions after deployment, build forms with complex logic in a web browser, and export form data to other tools. Data were cleaned according to the rules specified in the study protocol to ensure data integrity. Study data related to participating subjects and households, intervention (placement/replacement activity), mosquitoes collected, and laboratory analyses were cross-checked, identifying missing, incomplete, or suspect data submissions. These data were relayed to the site data manager to be resubmitted or corrected. Once data correction was complete, the data were verified and requested for analyses.

### Outcomes and statistical methods (Supplemental S1 statistical analyses plan).

The *primary analysis* of the study was intent-to-treat and included all the recruited subjects as per their treatment assignment. Study participants were excluded from the analyses either when they had no blood samples during the intervention period or when a household might have contributed more than two subjects. In the latter case, this occurred (rarely) when there was LTFU on the first recruited subject and a second subject was recruited as a replacement; only the subject with a longer follow-up period was used based on the per-protocol, allowing only one subject per household.

The main objective of the study was to demonstrate and quantify the PE of a SR product in reducing the incidence of malaria infection in a human cohort. Since the time to malaria in this study was measured and interval-censored, we compared the hazard rate, which is the instantaneous incidence rate, of the first-time infection and the overall infections between SR and placebo to address the study epidemiological objectives. The hazard rate is the main, and often the only, effect measure reported in many epidemiologic studies, including studies on malaria.^52–55^

The *primary hypothesis* on PE against first-time malaria infection was tested by comparing the hazard rates of the first-time malaria infection between active and placebo interventions. The complementary log–log (cloglog) regression model using $\log\left(-\log\left(1-\theta_{ijt}\right)\right)=\beta_{0t}+x_{ijt}^{t}\beta +z_{i}.$ ^56–59^ Here, $\theta_{ijt}$ is the discrete time hazard rate of subject j in cluster I at time $t;x_{\mathrm{ijt}}$contains visit (as a categorical predictor), the individual-level (age and gender), household-level (number of doors, open eaves *Y* or *N*, and wall type), and cluster-level (baseline incidence rate, cluster population size, and intervention group) covariates; and $z_{i}\text{∼ }N\left(0,\sigma^{2}\right)$ is the cluster-level random effect. Protective efficacy was estimated by $\left(1-\exp\left(\overset{⌢}{\beta }\right)\right)$ 100% with a 90% CI based on the Wald test, where $\overset{⌢}{\beta }$ is the estimated regression coefficient associated with the intervention group and exp($\overset{⌢}{\beta }$) is the estimated hazard ratio between the active and placebo. The null hypothesis that PE is 0 was tested by the Wald test $z=\overset{⌢}{\beta }/S$, where $\overset{⌢}{\beta }$ is the estimated standard error of $\overset{⌢}{\beta }$. It was concluded that active intervention reduces the first-time malaria hazard rate compared with placebo if $z<z_{0.05}=-1.645$; otherwise, the study would not have sufficient evidence to suggest that active intervention reduces the first-time malaria hazard rate compared with placebo at a one-sided significance level of 5%. The Kaplan–Meier curves on time to first infection per cluster were provided for the active and placebo arms, respectively.

The statistical analysis of the overall (total new) malaria infections detected in study subjects was similar to that used for analyzing the primary end point of the first-time malaria infection except that the aforementioned cloglog model has an additional term $z_{j\left(i\right)}$, which is the random effect at the individual level to account for the dependence among multiple malaria infections per individual. The same set of analyses as described earlier were performed in a subgroup analysis in clusters with nonzero baseline incidence rates and those clusters with entomology data.

The effects of active intervention on the *secondary entomological end points* were estimated using a negative binomial regression model, if applicable. Specifically, the *anopheline-landing rate* (surrogate “bite” based on HLC and indicator of human–vector contact) is defined as the number of mosquitos captured during a 12-hour evening interval (18:00 to 06:00 hours). The covariates in the model include the fixed effects of the intervention group, the interaction between treatment and location of collection (inside or outside), visit (as categorical), baseline incidence rate, baseline vector count, cluster population, and random effects for household nested within a cluster and for a cluster. The percentage change in the human-landing rate by intervention was estimated from the model. The frequencies and percentages of captured anophelines were also summarized by species. The set of covariates in the model for analyzing the *parity rate* are similar to the landing rate model, with an additional offset term for the daily landing rate. Because of data sparsity for the *sporozoite positivity rate* (> 99% of tested mosquitos were uninfected), no model-based analysis was performed and only summary statistics are provided. EIR is defined as the number of malaria-infective mosquito bites a person receives per unit time (typically annually) and calculated as EIR = sporozoite positivity rate × human biting rate. The observed data sparsity experienced with low sporozoite positivity also impacts the EIR results, and thus, only summary statistics are provided for EIR.

Besides the model-based analyses on the hazard rate of malaria infection, we also estimated the first-time incidence rate and the overall-time infection rate over the 2-year study period. The first-time incidence rate is calculated as the number of first-time infections divided by the person-time at risk for the first-time infection (the time taken to the first malaria infection, summed across all subjects in the study). The overall-time infection rate was calculated as the number of total malaria infections divided by the person-time at risk for malaria infection (the time taken to any new malaria infection, summed across all subjects during the whole study). The difference in the overall (total new) incidence rate per person-year between SR and placebo can be regarded as the total number of cases averted per person-year.

## RESULTS

### Protective effect against malaria infection (preplanned).

Trial outcomes show baseline covariates regarding subject, house construction, population, and baseline malaria incidence between the active and placebo arms were balanced at the individual-, household-, and cluster-level in the all 24 cluster analyses (Table 2). The intervention coverage rate, defined as the proportion of actually placed emanators over the total number required per household, ranged from 82.2% to 98.6% by cluster over the entire intervention period, with a mean application rate of 93.2–92.3% for the active and placebo arms, respectively. The percent of LLIN usage per household during the trial, defined as responding “yes” to the question “Did you use a bed net last night?” ranged from 14.6% to 99.8% in clusters that received active intervention and 17.4–99.8% in clusters that received placebo (Supplemental Information S2 LLIN Usage).

**Table 2:** Summary of baseline covariates for both spatial repellent (SR) and placebo treatment arms for the primary analysis

Individual level	SR (*n* = 665)	Placebo (*n* = 667)
Age (months) (mean ± SD (minimum, maximum))	34.0 ± 15.1 (6, 59)	34.2 ± 14.8 (6, 59)
Gender (% male subjects)	54.1%	51.1%
Household level		
	SR (*n* = 665)	Placebo (*n* = 667)
House wall type (wood %)	91.6%	94.0%
Open eaves (yes %)	98.0%	99.6%
No. of doors (mean ± SD (minimum, maximum))	2.06 ± 0.38 (1, 4)	2.06 ± 0.30 (0, 4)
Cluster level		
	SR (*n* = 12)	Placebo (*n* = 12)
Cluster population (mean ± SD (minimum, maximum))	694.3 ± 59.2 (624, 820	730.8 ± 129.0 (616,1117
Baseline incidence rate per person-year (mean ± SD (minimum, maximum))	0.096 ± 0.115 (0, 0.426	0.089 ± 0.088 (0, 0.265
Baseline overall (total new) infection incidence per person-year (mean ± SD (minimum, maximum))	0.094 ± 0.111 (0, 0.412	0.089 ± 0.087 (0, 0.261

SR = spatial repellent.

There were 134 first-time infections among 665 subjects in active intervention households and 164 first-time infections in 667 subjects in placebo-control households, with a 27.7% decrease in the first-time malaria hazard risk using active compared with placebo (90% CI: −21.3%, 56.9%) (Table 3). The 27.7% PE was not statistically significant at the 5% one-sided significance level (*P* = 0.151). The estimated PE of active intervention against overall (total) malaria infections (first and subsequent) was 31.3% (90% CI: −10.8, 57.4%) with a one-sided *P*-value of 0.098 (Table 3). Investigation of potential shift in parasite species infection frequency (*P. falciparum* versus *Plasmodium vivax*) between the active and placebo arms will be reported in subsequent publications.

**Table 3:** Summary of first-time and overall (total new) malaria incidence during the study

	First-time infection			Overall (total new) infections		
	All clusters	Subgroup 1*	Subgroup 2†	All clusters	Subgroup 1*	Subgroup 2†
	SR: placebo					
No. of clusters	12:12	10:9	6:6	12:12	10:9	6:6
No. of households	665:667	557:500	335:335	665:667	557:500	335:335
No. of infections	134:164	130:158	93:140	253:439	249:433	196:408
Person years	1079:1032	873:722	506:444	1216:1216	1015:909	609:607
Incidence rate (per person-year)	0.124:0.159	0.149:0.219	0.184:0.315	0.208:0.361	0.245:0.476	0.322:0.672
Hazard ratio (90% CI)	0.723 (0.431,1.213)	0.64 (0.299, 1.367)	0.447 (0.302, 0.664)	0.687 (0.426,1.108)	0.591 (0.382, 0.914)	0.344 (0.233, 0.508)
Protective efficacy (%) (90% CI)	27.7 (−21.3, 56.9)	33.3 (−7.8, 58.7)	55.3 (33.6, 69.9)	31.3 (−10.8, 57.4)	40.9 (8.61, 61.8)	65.6 (49.2, 76.7)
One-sided *P*-value	0.151	0.083	0.0004	0.098	0.0236	< 0.0001

SR = spatial repellent.

* Subgroup 1: clusters with nonzero incidence during intervention.

† Subgroup 2: clusters with entomology data collected (moderate to high risk of infection).

**Figure 5.**
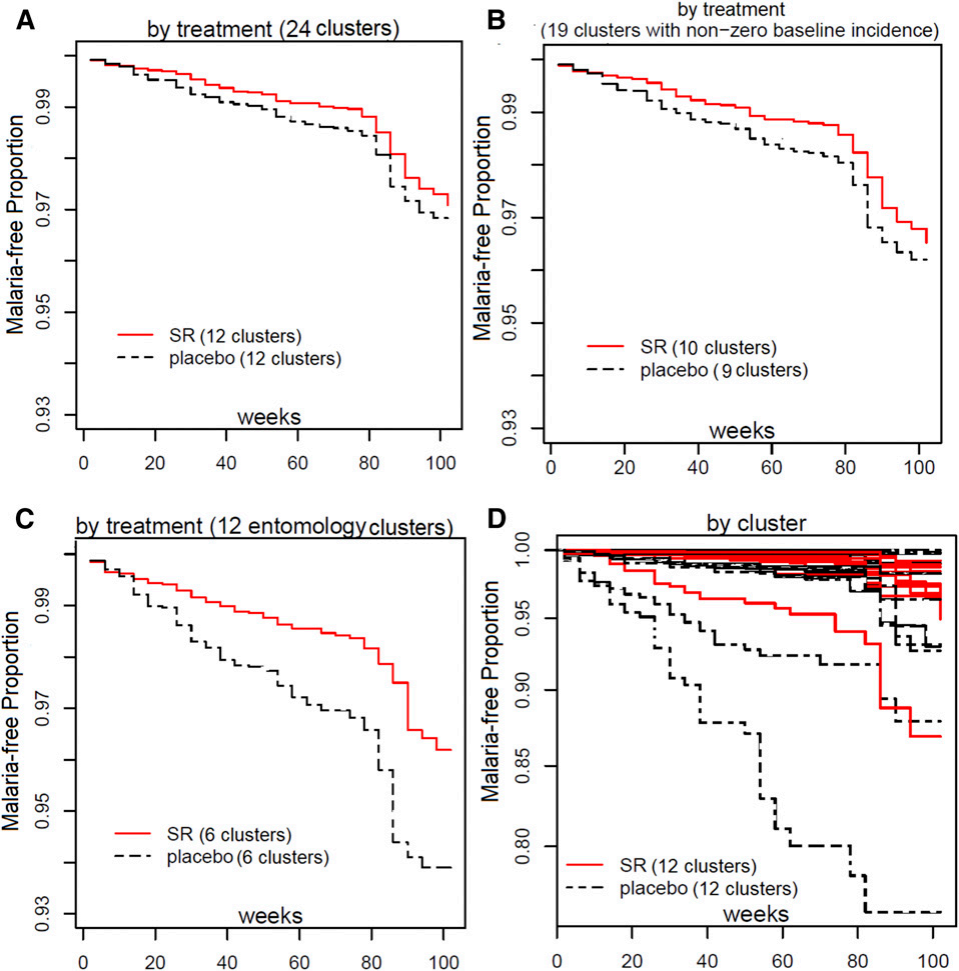
Kaplan-Meier curves by treatment (all clusters [**A**]; subset analysis [**B** and **C**]) and by cluster (**D**).

A total of 164 first-time malaria infections occurred during approximately 1,032 person-years at risk in study participants whose households were given placebo, with a calculated incidence density of 0.159 infections/person-year (Table 3, Figure 5).^60^ By contrast, 134 total malaria attacks occurred in transfluthrin-active households with approximately 1,079 person-years at risk, resulting in a calculated 0.124 infections/person-year. A cumulative 439 malaria infections (first-time and subsequent) during approximately 1,216 person-years at risk in participants with households provided placebo produced an incidence density of 0.361 infections/person-year. By contrast, 253 total (accumulative) malaria attacks occurred among participants living in active intervention households with approximately 1,216 person-years at risk, equaling 0.209 infections/person-year (Table 3).

### Subgroup analyses of protective effect against malaria infection among nonzero incidence and entomology clusters (not preplanned).

Among the 24 clusters, there were five clusters with zero baseline incidence having had zero to very low incidence rates during the intervention. The first subgroup analysis used the 19 clusters with nonzero incidence rates as the remaining five clusters may have been excluded from the intervention if baseline analysis had been performed before randomization. Excluding these five clusters, the estimated PE for overall (total new) infections in the remaining subgroup of 19 clusters with nonzero baseline incidence rates was 40.9% (90% CI: 8.61, 61.8%), resulting in a one-sided *P*-value of 0.0236 (Table 3). The second subgroup analyses included incidence from the 12 clusters having routine entomology collection data (i.e., mean anopheline-landing rate/person), and where the average baseline incidence was approximately 4-fold greater than the other study clusters. The PE using active intervention against time to first-event and overall (total new) malaria infections in this subgroup was 55.3% (90% CI: 33.6, 69.9; *P*-value < 0.0004) and 66% (90% CI: 49.2%, 76.7%; *P*-value < 0.0001), respectively (Table 3). The data indicate the baseline covariates (subject, house construction, population, and baseline malaria incidence) between the active and placebo arms were balanced at the individual, household, and cluster levels in the 19 nonzero incidence and 12 entomology cluster subgroup analyses (not shown).

### Effects on entomological end points (preplanned).

Results presented are summary outcomes on aggregated anopheline species captured using HLCs. Detailed data analyses on effects of this intervention on entomology measures are forthcoming in subsequent publications, to include temporal change in species-specific anopheline vector composition over the trial period, relative abundance between treatment arms by species, and the HLC of non-anophelines (culicine mosquitoes).

#### Anopheline-landing rates.

**Figure 6.**
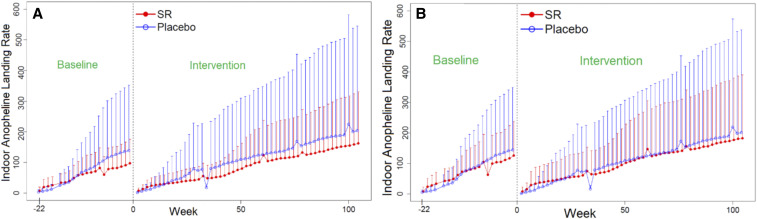
Mean (+SD) cumulative biweekly indoor (**A**) and outdoor (**B**) anopheline human-landing catch averaged over 20–24 households per treatment arm—spatial repellent intervention and placebo, respectively.

A total of 52 weeks of HLCs were performed within a subset of 12 clusters during the intervention period. Results based on morphological species identification detected 19 putative malaria vector species showing spatial and temporal variations across monitored clusters. The most common anopheline species (s.s. or s.l.) attracted to humans included *Anopheles aconitus*, *An. annularis*, *An. barbirostris*, *An. flavirostris*, *An. kochi*, *An. maculatus*, *An. subpictus*, *An. sundaicus*, *An. tessellatus*, and *An. vagus* (Supplemental Information S3 Anopheline Frequency Summary). The cumulative indoor (*n* = 8,780) and outdoor (*n* = 9,207) anopheline-landing rates across both baseline and intervention sampling periods are shown in Figure 6. The reduction in the anopheline attack rate on collectors positioned indoors and outdoors, at sentinel households with active intervention compared with placebo houses, was not statistically significant: 16.4% (95% CI: = −75.2%, 182.7%; *P* = 0.774) and 11.3% (95% CI: = −73.7%, 199.4%; *P* = 0.847), respectively (Table 4).

**Table 4:** Intervention effect on anopheline mosquito-landing rates by collection location

Location*	Spatial repellent (mean ± SD) †	Placebo (mean ± SD) †	% Change (95% CI) SR vs. placebo †	Two-sided *P*-value
Indoor	3.14 ± 5.84	3.97 ± 8.73	−16.4 (−75.2, 182.7)	0.774
Outdoor	3.54 ± 7.13	3.93 ± 8.64	−11.3 (−73.7, 199.4)	0.847

* Position at each human-landing catch (HLC) sentinel house where sample was captured (indoor = near center of house; outdoor = on verandah ∼1 m from the edge of exterior wall.

† Human-landing rate based on 12-hour collection per person.

#### Sporozoite positivity rate and entomological inoculation rate (EIR).

A total of 11,928 and 17,986 anopheline samples from baseline and intervention follow-up periods, respectively, were processed for malaria sporozoites. The frequency of sporozoite-positive anophelines is provided in Table 5. Only *P. falciparum* and *P. vivax* infections were detected in captured mosquitoes. During the baseline and intervention periods, the sporozoite rate was less than 0.5% for both treatment and placebo arms. Data sparsity regarding comparison of sporozoite rates precluded inferential statistical analyses. The EIR was < 1 bite per year in both treatment arms during the baseline and intervention periods.

**Table 5:** Frequency of anopheline (all species) sporozoite infection status for both spatial repellent (SR) and placebo treatment arms

Treatment allocation	*Plasmodium falciparum*	*Plasmodium vivax*.	Indeterminate*	Noninfected	Sporozoite positivity rate†
Baseline					
SR	12	9	0	4,706	0.4%
Placebo	12	9	0	6,244	0.3%
Intervention					
SR	3	8	0	8,130	0.1%
Placebo	6	1	1	9,615	0.1%

SR = spatial repellent.

* Indeterminate defined as *Plasmodium*-positive sample but parasite species not identifiable.

† Sporozoite positivity rate = [(Pf + Pv)/(Pf + Pv + indeterminate + noninfected)] x 100%.

#### Parity rate (age-grading).

A total of 15,828 anopheline samples were dissected for parity status during the trial period (Table 6). The proportion of females categorized as “older,” combining parous and gravid states (mosquitoes with advanced ovarian follicle development as evidence of a recent blood meal), and those “younger,” as nulliparous (non–blood-fed) and more recently emerged, was compared between active and placebo treatments for the 12 clusters with entomological monitoring. Overall, transfluthrin-active emanators proportionally increased nulliparity in the sampled anopheline populations compared with placebo for both indoor and outdoor locations (Table 6).

**Table 6:** Spatial repellent (SR) intervention effect on parity and nulliparity rates (all anopheline species)

	HLC location*	SR (mean ± SD)†	Placebo (mean ± SD)	% Change (95% CI) SR vs. placebo	Two-sided *P*-value
Parous	Indoor (*n* = 5,784)	0.41 ± 0.44 (*n* = 2,537)	0.41 ± 0.45 (*n* = 3,247)	−10.2 (−62.1, 113.2)	0.808
	Outdoor (*n* = 6,193)	0.40 ± 0.44 (*n* = 2,907)	0.43 ± 0.45 (*n* = 3,286)	−25.9 (−68.8, 75.6)	0.495
Nulliparous	Indoor (*n* = 1,873)	0.16 ± 0.29 (*n* = 994)	0.12 ± 0.26 (*n* = 879)	58.3 (−37.0, 298.0)	0.329
	Outdoor (*n* = 1,978)	0.17 ± 0.30 (*n* = 1,126)	0.11 ± 0.25 (*n* = 852)	54.9 (−37.6, 284.3)	0.346

* Position of collect/or conducting human-landing catch (HLC) (indoor = near center of house; outdoor = on house verandah ∼1 m from the edge of exterior wall).

† Mean and SD are descriptive statistics, and % change was obtained from fitting negative binomial models.

#### Insecticide susceptibility.

A total of 5,091 adult female anophelines (chemical and control bioassays) were evaluated for susceptibility: 700 samples during baseline, 1,805 during intervention, and 2,586 post-intervention. *Anopheles vagus* was the most widely distributed and tested anopheline (56%), followed by *An. sundaicus* (12.6%) and *An. subpictus*, *An. barbirostris*, *An. kochi*, *An. aconitus*, *An. maculatus*, and *An. tessellatus* (proportionally, all < 10%). In the CDC bottle bioassay, baseline, intervention, and post-intervention tests using permethrin (21.5ng/mL) showed 100% knockdown between 15 and 30 minutes exposure and within the recommended diagnostic time. In the WHO tube bioassay with rare exception, there was 100% 24-hour mortality to permethrin 0.8% following 60-minute exposure. Based on the assumption of predominately pyrethroid-susceptible wild populations of *Anopheles* species present in trial sites, there was no conclusive evidence of the development of phenotypic resistance to pyrethroid class chemicals between pre- and post-intervention periods. Interestingly, in several clusters during the post-intervention phase, there were indications of reduced permethrin susceptibility (WHO bioassay) in a few *An. barbirostris* populations to permethrin. Follow-up investigations provided inconsistent results; thus, the confirmation that these focal populations showed low levels of resistance could not be verified. The determination of presence or absence of target-site mechanisms for resistance will be reported separately.

### Adverse events and SAEs.

Using government clinic health records, a total of 523 AEs were reported from nonparticipants, whereas 144 AEs were reported from the intervention study cohort captured by the study team. Of subject AEs, 52 were reported from participants in the active intervention arm and 92 from placebo clusters. General respiratory complaints were most common for all reported AEs, followed by general fever. There were six total SAEs reported in the overall study cohort during the follow-up: one death due to suspected brain infection, one death from respiratory infection, one death from malaria with concomitant bacterial or viral infection, one death by drowning, and two deaths of unknown cause.

### Intervention quality control.

A total of 180 unused emanator samples were analyzed in 2017. Samples were taken from 12 different active and placebo clusters with manufacturing dates ranging from April 2016 to November 2016 (15-month- to 8-month-old samples). All sampled placebo interventions (*n* = 48) were found to be absent for transfluthrin. The average transfluthrin quantity from all sampled active emanators (*n* = 132) was 54.3 mg, which was within the specification range (55.0 mg ± 2.75 mg).

## DISCUSSION

Malaria remains a significant global public health burden despite recent progress in reducing transmission rates.^61^ The primary objective of this large-scale RCT conducted on Sumba Island, Indonesia, was to demonstrate and quantify the PE of a passive emanating SR intervention (transfluthrin treated), for reducing malaria incidence (transmission) in humans. Such epidemiological evidence of health impact is a fundamental requirement in the critical path of development of new vector control intervention classes being assessed by the WHO VCAG.^16^ Sumba Island, Indonesia, represents a group of malaria-endemic settings where SRs are intended to be implemented if/when a policy recommendation is endorsed as Sumba does not currently conduct routine IRS, LLINs were only recently distributed, and there is a range of anopheline biting habits by local vectors, including early-evening and/or outdoor biting.

The primary per-protocol analyses provide an estimate of 27% PE against malaria infection, which is near the targeted efficacy of 30% used in the power calculations. This effect, however, was not statistically significantly protective at the one-sided 5% significance level. There were 298 first-time infections, in contrast to an expected number of at least 417 assumed in the sample size calculations, thus resulting in the trial being underpowered. The demonstration of spatial repellency PE (66%) against total new infections in the subset of 12 clusters used for entomological measures, which had an approximately 4-fold greater malaria infection incidence during intervention, indicates health impact where risk of malaria infection was greatest (i.e., locations with highest vector-landing densities). Outcomes reported here are promising for malaria control, despite the study being unable to demonstrate clear, statistically PE on primary analysis, with data anticipated to be included in meta-analyses with other clinical trials evaluating SRs.

This study highlights three primary challenges for consideration in future SR trials as well for new vector control intervention classes more broadly: 1) having “adaptive” study designs, especially for evaluation of interventions in low to moderate malaria-transmission settings and/or settings with inherently large cluster-to-cluster variance on transmission intensity, 2) defining and identifying the “key” entomological correlates of protection, and 3) ensuring reliability and feasibility in AE/SAE reporting for accurate safety assessment. Regarding adaptive study designs, our assumptions for power and sample size calculations were based on a previous proof-of-concept study that took place in an entirely coastal location but was spatially very near (bordering) the current study area.^19^ The study villages, both coastal and further inland, were selected with the assumption that all clusters delineated from these villages would experience similar levels of malaria transmission during the intervention period; however, this was not the case during the trial. As in all epidemiological trials, underlying assumptions of incidence used for power and sample size calculations can vary from actual incidence during intervention follow-up. Baseline data analyses indicated zero incidence for five of the 24 selected study clusters with a 5-fold higher between-cluster variability than assumed in the original sample size calculation. The five clusters with zero baseline incidence (two allocated to receive active and three allocated to receive placebo) also had either zero or low incidence rates during intervention and ranked in the top eight among the 24 clusters in terms of lowest in intervention incidence (data not shown).

In retrospect, preplanned interim and final analyses of the baseline data would have allowed for consideration in adjustment of the study design by filtering out the zero-incidence clusters, or increasing the study duration (time of follow-up), the number of subjects recruited per cluster, or the number of clusters to better satisfy requirements of statistical power and capture the necessary number of outcome events (infections). However, baseline incidence was measured only for verifying hierarchical stratification and used as a covariate in statistical analyses of PE. This, therefore, resulted in 1) the original sample size not accommodating for interim and final analyses of baseline data, 2) timelines of grant period not being projected and assured to include lag-time for baseline data analyses and outcome assessment, and perhaps most importantly 3) baseline incidence analyses not being explored early to determine variations in sample size/cluster number adjustments required to achieve sufficient study power and subsequent study site viability.

Given the updated baseline incidence rate of 0.131 per person-year and 97.1% CV, to maintain 80% power with the originally assumed 30% PE, 100 clusters per arm with 144 households (i.e., subjects) would have been required to be recruited to collect 5,550 first-time malaria events—an unmanageable scale that could not have been supported because of geographical, logistical, and funding constraints. Moreover, increasing the follow-up time to collect more first-time malaria events would have neither counterbalanced the large variability nor the longer duration required to collect the additional events because of the seasonally influenced low to moderate malaria transmission among clusters. For this reason, when planning future trials in low or moderate endemic settings, investigators should consider including a greater number of clusters per arm and/or building into the statistical analyses plan interim and final analyses of *baseline* incidence with predetermined study design adaptations, to include down selection of clusters with predetermined incidence thresholds. This may become most relevant when considering proof of efficacy of an intervention in low transmission settings as a component for malaria elimination goals. Early exercises of the impact of a lower-than-assumed incidence (or greater CV) during study planning should be explored among investigators, industry, and funding partners to ensure that the study area context, intervention manufacturing, program period, and/or funding can sufficiently meet the demands of power requirements if adjustments are to be made once the study begins. Stakeholders should also discuss supporting and adopting adaptive designs, thereby allowing decision-making after a planned interim analysis of *intervention* data, to either stop the trial for futility or to continue the trial with adjusted design parameters, such as sample size.^62,63^ Perhaps, just as important in the context of vector control, although RCTs are rated as high-quality evidence,^64^ considerations to RCT alternatives are prudent in the trial planning phase as these may offer assurances of adequate data rigor while balancing cost and time constraints of traditional RCTs.^65,66^ Alternative study designs include large observational studies for detecting population-level effects using analytical cross-sectional studies or operational program-based evidence; the latter perhaps especially for interventions containing an existing registered chemical active ingredient (i.e., meets human safety thresholds) and where the intervention is implemented in pilot trials and impact monitored through case reports, as compared with a contemporaneous control group.^66^

Albeit the study was not powered for entomology, the inclusion of entomological end points in the Sumba RCT was, in part, to help understand and validate the intervention’s mode of action for the VCAG claim of a health impact through a reduction in human–vector contact. SR chemicals may cause initial knockdown and mortality by exposure to toxic doses at close range to the active ingredient or a delayed kill through behavioral avoidance response (i.e., blood-feeding inhibition), through exposure to sublethal doses at distances further away from the stimulus source. Therefore, entomological measurements for detecting reductions in human-landing density in clusters with active intervention, and a possible change in anopheline age structure (parity rate) indicating reduced daily survival of mosquitoes in clusters with intervention, was built into this study. In addition, a reduction in sporozoite infection rates, because of lower blood-feeding success, and/or an inability to survive the required time interval (parasite incubation period) to become infective (transmissible) from a vector to a host represent other end point measures of repellency impact. A causal relationship with one, several, or all of these end points would allow future trials evaluating non-inferiority of a “second-in-class” SR to integrate entomological measures only to predict PE and provide assurances of meeting minimum thresholds of acceptance for public health use. The value arguably is a potential reduction in cost and time, which subsequently could further incentivize industry R&D with the goal of increasing varying types of quality and efficacious SR products available for global implementation.

Sporozoite positivity rates and EIR estimates from the current trial were low, with a total of 42 of 11,650 (0.4%) anophelines tested during baseline (10 months) and 19 of 17,971 (0.1%) found infected during intervention (24 months). These findings are not unusual or unexpected in low to moderate malaria-transmission settings.^35^ As an example, the previous Sumba study (using metofluthrin coils) reported just 15 of 1,825 (0.8%) HLC anopheline samples were sporozoite infected.^19^ The challenges for assessing sporozoite infectivity and using EIR as a measure of intervention effect in such settings should be factored into study preplanning to carefully balance cost of sample processing with potential useful information gained.

These study findings report a 16.4% numerical reduction in indoor-landing mosquitoes exposed to active treatment compared with placebo-control. Just as important was an observed numerical reduction (11%) on outdoor-landing collections on the exposed verandah of sentinel houses, as Sumba residents often sleep on the verandah without the protection of a bed net. It is noted, however, that both indoor and outdoor HLC outcomes showed wide CIs that overlap zero, making results inconclusive; nevertheless, the intervention showed impact against malaria in these clusters. A previous proof-of-concept study on Sumba that evaluated a metofluthrin-active coil resulted in a significant 32% reduction in *An. sundaicus* indoor-landing rates as compared with a blank coil control.^19^ The minimal reduction in the human-landing rate in the current RCT may be the result of species-specific effects by active ingredient (i.e., metofluthrin versus transfluthrin), but differences in HLC outcomes of the two studies on Sumba should not be interpreted as the transfluthrin intervention being less efficacious than a coil. The greater complexity of anopheline diversity may likely have contributed to the inconclusive HLC outcomes based on aggregated anopheline data. It is expected similar results may occur in future trials that are conducted in settings with diverse vector populations and/or as cluster size increases, thereby increasing the probability of greater habitat diversity.

Specifically, the previous Sumba study was smaller in scale, and *An. sundaicus* was the overwhelmingly predominate human-feeding anopheline collected, whereas in the current study, this species was relatively uncommon during the intervention trial. The range of ecologies (coastal plains to upland forested hills) varied greatly across the 12 clusters used for entomology collections in this trial compared with that of the earlier Sumba study where collections were confined to only four adjoining clusters having similar coastal environments. This spatial variability in ecology contributed to a broader range of species diversity. We report 19 species/group (species identified), each species with different bionomic and behavioral characteristics (e.g., biting habits and host preferences). Perhaps most important, the efficacy of an intervention is related to epidemiological outcomes (reduced risk of infection); HLC outcomes reported from the current study were inconclusive, but nevertheless showed impact against malaria.

Insecticide susceptibility monitoring was integrated into the study to characterize the wild-type anopheline populations to estimate PE against resistant vector populations, if resistance was expressed, and monitor changes in susceptibility due to continuous exposure to transfluthrin. Our data indicate that continual use of transfluthrin as an SR during a consecutive 24-month period did not result in a change in phenotypic response to permethrin. Although we are confident in the findings presented, there were limitations in sampling from multiple sites and handling numerous species during pre-, within-, and post-intervention periods. This may have compromised the study’s ability to obtain a more robust and definitive profile of susceptibility background and detection of any shift in phenotypic (and molecular) frequencies.

The third “lesson learned” from the current study relates to AE and SAE monitoring. Reports of intervention safety in this study should take into account possible limitations of the data collection. The DSMB was provided with clinic attendance data from January 2015 to December 2018 for upper respiratory infection, pneumonia, and malaria, as classified by month, health center, and village within health center coverage. Although not gathered as part of the study, the DSMB was keenly interested in these data because of the surprisingly low number of illnesses/deaths reported from the study population during the 24-month intervention period. For some periods, particularly for malaria infection, the case data were missing or the health center totals were not available at the village level. Of the 13 villages represented in the dataset, from which study clusters were formed, all but four comprised clusters (or parts of clusters) from both study intervention arms (i.e., allocated active or placebo); therefore, it was not possible to infer the allocation status of the corresponding cases. For this reason, the DSMB was unable to make a reliable assessment of any possible association between active intervention and clinic attendance for those reported health conditions. The respiratory illnesses in the clinic data are more reflective of the magnitude of numbers of cases one would expect from this population; however, the DSMB was again unable to parse them into test and control clusters because of the reasons aforementioned, and therefore, the data were of little use for monitoring purposes. The number of SAEs reported, a total of six, is well below the expected in a study population of this size. The WHO estimates the crude death rate in Indonesia to be approximately 6.2 per 1,000 per year.^67^ Based on this, for a population of 1,296 children enrolled (protocol sample size), the probability of having zero deaths in a year is less than one in 1,000. Although open commemoration of deaths is commonly practiced in the study area, it seems more likely deaths were not completely reported/recorded as SAEs.

Overall, the point to apply for future SR trials (and perhaps other trials of new vector control interventions) is to improve the mechanism for capturing and communicating AEs and SAEs before the initiation of the study to better ensure reliable reporting and regular monitoring for unusual numbers of complaints. If these studies are regarded as a clinical trial with a placebo arm that requires comparative AE and SAE data, the scale could cause failure in safety assessment simply on the mass of data, however imperfectly collected. Any safety signal detected would most likely be due to bias in reporting and/or collection bias and unrelated with the investigational intervention. Trials evaluating an SR intervention with a registered active ingredient (i.e., a chemical meeting regulatory approvals for acceptable levels of human risk), such as transfluthrin, could focus on a small number of complaints that might be expected because of inhalation (i.e., volatilization) and develop a monitoring scheme that collects consistent data across the study population. Clinic-based complaints of respiratory illness such as pneumonia and asthma might be possible, but the variability in their method of collection (clinic or survey) will likely be highly dependent on study site infrastructure.

In conclusion, although more evidence will be required to determine whether SRs can serve as a viable malaria control intervention, both the primary and secondary results of this Sumba Island trial have generated valuable data and observations that can contribute to the overall assessment and improvement of testing protocols of an SR intervention class. The VCAG has recommended that data from at least one additional trial evaluating the SR be generated,^68^ and once available, the panel will be able to assess the available evidence for judging public health value. If the crude estimate of PE shown here, near 30%, is replicated in future statistically robust cluster-randomized trials, the intervention prototype evaluated in this RCT would approximate the benefit associated with LLINs.^69^ Perhaps, just as important, the number of cases averted indicates 361 expected cases per 1,000 persons per year (0.361 × 1,000 ×1) without the SR and 152 less cases expected when using the active intervention ([0.361–0.209] × 1,000 × 1). The cost savings of averting these 152 less cases per 1,000 person-years is an important consideration for strengthening health systems. These results have encouraged further and substantial investment to validate SR efficacy through larger RCTs, including an investigation of possible vector diversionary effects on human health (i.e., greater than expected malaria incidence in households near intervention not receiving active product) and evaluation of the optimal delivery systems for humanitarian assistance use case scenarios.^70^

## Supplemental informations

Supplemental materials

## References

[1] Kennedy JS, 1947 The excitant and repellent effects on mosquitos of sub-lethal contacts with DDT. Bull Entomol Res 37: 593–607. 20287803 10.1017/s0007485300030091

[2] Muirhead-Thomson RC, 1951 Mosquito Behaviour in Relation to Malaria Transmission and Control in the Tropics. London, United Kingdom: Edward Arnold & Co, 219.

[3] De Zulueta J Cullen JR Smith A, 1963 Deterrent effect of insecticides on malaria vectors. Nature 200: 860–862. 14096060 10.1038/200860a0

[4] Dethier VG Browne BL Smith CN, 1960 The designation of chemicals in terms of the responses they elicit from insects. J Econ Entomol 53: 134–136. 10.1603/029.102.0606 20069831

[5] Miller JR Siegert PY Amimo FA Walker ED, 2009 Designation of chemicals in terms of the locomotor responses they elicit from insects: an update of Dethier et al. (1960). J Econ Entomol 102: 2056–2060. 20069831 10.1603/029.102.0606

[6] Roberts DR Alecrim WD Hshieh P Grieco JP Bangs M Andre RG Chareonviriphap T, 2000 A probability model of vector behavior: effects of DDT repellency, irritancy, and toxicity in malaria control. J Vector Ecol 25: 48–61. 10925797

[7] WHO, Pesticide Evaluation Scheme, 2013 Guidelines for Efficacy Testing of Spatial Repellents. Geneva, Switzerland: World Health Organization.

[8] Grieco JP Achee NL Sardelis MR Chauhan KR Roberts DR, 2005 A novel high-throughput screening system to evaluate the behavioral response of adult mosquitoes to chemicals. J Am Mosq Control Assoc 21: 404–411. 16506566 10.2987/8756-971X(2006)21[404:ANHSST]2.0.CO;2

[9] Achee NL Sardelis MR Dusfour I Chauhan KR Grieco JP, 2009 Characterization of spatial repellent, contact irritant, and toxicant chemical actions of standard vector control compounds. J Am Mosq Control Assoc 25: 156–167. 19653497 10.2987/08-5831.1

[10] Ogoma SB Ngonyani H Simfukwe ET Mseka A Moore J Maia MF Moore SJ Lorenz LM, 2014 The mode of action of spatial repellents and their impact on vectorial capacity of *Anopheles gambiae* sensu stricto. PLoS One 9: e110433. 25485850 10.1371/journal.pone.0110433 PMC4259296

[11] Ogoma SB 2014 An experimental hut study to quantify the effect of DDT and airborne pyrethroids on entomological parameters of malaria transmission. Malar J 13: 131. 24693934 10.1186/1475-2875-13-131 PMC4230423

[12] Grieco JP Achee NL Chareonviriyaphap T Suwonkerd W Chauhan K Sardelis MR Roberts DR, 2007 A new classification system for the actions of IRS chemicals traditionally used for malaria control. PLoS One 2: e716. 17684562 10.1371/journal.pone.0000716 PMC1934935

[13] U.S EPA Office of Pesticide Programs, 2018 Conventional New Chemical Registration Decisions - Completed FY 2018. Arlington, VA: U.S. EPA Office of Pesticide Programs.

[14] Norris E Coats J, 2017 Current and future repellent technologies: the potential of spatial repellents and their place in mosquito-borne disease control. Int J Environ Res Public Health 14: 124. 10.3390/ijerph14020124 PMC5334678 28146066

[15] Debboun M Frances SP Strickman D, 2006 Insect Repellents: Principles, Methods, and Uses. Boca Raton, FL: CRC Press, 495.

[16] WHO, 2017 The Evaluation Process for Vector Control Products. Geneva, Switzerland: World Health Organization.

[17] Maia MF Kliner M Richardson M Lengeler C Moore SJ, 2018 Mosquito repellents for malaria prevention. Cochrane Database Syst Rev 2: CD011595. 29405263 10.1002/14651858.CD011595.pub2 PMC5815492

[18] Hill N Zhou HN Wang P Guo X Carneiro I Moore SJ, 2014 A household randomized, controlled trial of the efficacy of 0.03% transfluthrin coils alone and in combination with long-lasting insecticidal nets on the incidence of *Plasmodium falciparum* and *Plasmodium vivax* malaria in western Yunnan province, China. Malar J 13: 208. 24885993 10.1186/1475-2875-13-208 PMC4057598

[19] Syafruddin D 2014 Impact of a spatial repellent on malaria incidence in two villages in Sumba, Indonesia. Am J Trop Med Hyg 91: 1079–1087. 25311699 10.4269/ajtmh.13-0735 PMC4257627

[20] WHO-Department of Control of Neglected Tropical Diseases, 2017 How to Design Vector Control Efficacy Trials. Guidance on Phase III Vector Control Field Trial Design. Geneva, Switzerland: World Health Organization, 62.

[21] Ogoma SB Moore SJ Maia MF, 2012 A systematic review of mosquito coils and passive emanators: defining recommendations for spatial repellency testing methodologies. Parasit Vectors 5: 287. 23216844 10.1186/1756-3305-5-287 PMC3549831

[22] Achee NL Grieco JP, 2012 Is it time to formally recognize spatial repellency for disease prevention? Outlooks Pest Management 23: 283–286.

[23] World Health Organization, 2017 Global Vector Control Response 2017–2030. Geneva, Switzerland: WHO.

[24] Pates H Curtis C, 2005 Mosquito behavior and vector control. Annu Rev Entomol 50: 53–70. 15355233 10.1146/annurev.ento.50.071803.130439

[25] Durnez L Coosemans M, 2013 Residual Transmission of Malaria: An Old Issue for New Approaches. Anonymous Anopheles Mosquitoes - New Insights into Malaria Vectors. London, United Kingdom: IntechOpen.

[26] Harvey SA Lam Y Martin NA Olórtegui MP, 2017 Multiple entries and exits and other complex human patterns of insecticide-treated net use: a possible contributor to residual malaria transmission? Malar J 16: 265. 28673285 10.1186/s12936-017-1918-5 PMC5496366

[27] Monroe A Asamoah O Lam Y Koenker H Psychas P Lynch M Ricotta E Hornston S Berman A Harvey SA, 2015 Outdoor-sleeping and other night-time activities in northern Ghana: implications for residual transmission and malaria prevention. Malar J 14: 35. 25627277 10.1186/s12936-015-0543-4 PMC4320825

[28] World Health Organization, 2013 Malaria Control in Humanitarian Emergencies – An Inter-Agency Field Handbook, 2nd edition Geneva, Switzerland: WHO.

[29] Achee NL 2012 Spatial repellents: from discovery and development to evidence-based validation. Malar J 11: 164. 22583679 10.1186/1475-2875-11-164 PMC3453515

[30] Hemingway J Shretta R Wells TNC Bell D Djimdé AA Achee N Qi G, 2016 Tools and strategies for malaria control and elimination: what do we need to achieve a grand convergence in malaria? PLoS Biol 14: e1002380. 26934361 10.1371/journal.pbio.1002380 PMC4774904

[31] World Health Organization, 2019 Guidelines for Malaria Vector Control. Geneva, Switzerland: World Health Organization, 1–171. Available at: https://www.who.int/malaria/publications/atoz/9789241550499/en/. Accessed March 16, 2020.

[32] Center for Drug Evaluation and Research, Center for Biologics Evaluation and Research, 2018 E6(R2) Good Clinical Practice: Integrated Addendum to ICH E6(R1). Silver Spring, MD: US Food and Drug Administration.

[33] OECD, 1998 OECD Series on Principles of Good Laboratory Practice (GLP) and Compliance Monitoring. Available at: www.oecd.org/chemicalsafety/testing/oecdseriesonprinciplesofgoodlaboratorypracticeglpandcompliancemonitoring.htm. Accessed July 10, 2019.

[34] Badan Pusat Statistik, 2010 2010 Population Census - Indonesia. Available at: http://sp2010.bps.go.id/. Accessed July 10, 2019.

[35] Elyazar IRF Sinka ME Gething PW Tarmidzi SN Surya A Kusriastuti R Winarno Baird JK Hay SI Bangs MJ, 2013 The distribution and bionomics of *Anopheles* malaria vector mosquitoes in Indonesia. Rollinson D, ed. Advances in Parasitology. Amsterdam, The Netherlands: Elsevier Ltd, 173–266. 10.1016/B978-0-12-407705-8.00003-3 23876873

[36] Barbara KA Sukowati S Rusmiarto S Susapto D Bangs MJ Kinzer MH, 2011 Survey of *Anopheles* mosquitoes (Diptera:Culicidae) in West Sumba district, Indonesia. Southeast Asian J Trop Med Public Health 42: 71–82. 21323168

[37] Lievens M Aponte JJ Williamson J Mmbando B Mohamed A Bejon P Leach A, 2011 Statistical methodology for the evaluation of vaccine efficacy in a phase III multi-centre trial of the RTS, S/AS01 malaria vaccine in African children. Malar J 10: 222. 21816030 10.1186/1475-2875-10-222 PMC3167766

[38] LaRue N 2014 Comparison of quantitative and qualitative tests for glucose-6-phosphate dehydrogenase deficiency. Am J Trop Med Hyg 91: 854–861. 25071003 10.4269/ajtmh.14-0194 PMC4183416

[39] Nkrumah B Nguah SB Sarpong N Dekker D Idriss A May J Adu-Sarkodie Y, 2011 Hemoglobin estimation by the HemoCue® portable hemoglobin photometer in a resource poor setting. BMC Clin Pathol 11: 5. 21510885 10.1186/1472-6890-11-5 PMC3095531

[40] WHO Specifications and Evaluations for Public Health Pesticides, 2016 Transfluthrin. Geneva, Switzerland: World Health Organization.

[41] United States Environmental Protection Agency, 2018 Registration Decision for the New Active Ingredient, Transfluthrin. Washington, DC: US EPA.

[42] Echeverry DF 2016 Human malaria diagnosis using a single-step direct-PCR based on the *Plasmodium* cytochrome oxidase III gene. Malar J 15: 128. 26928594 10.1186/s12936-016-1185-x PMC4772515

[43] World Health Organization & UNICEF/UNDP/World Bank/WHO Special Programme for Research and Training in Tropical Diseases, 2015 Microscopy for the Detection, Identification and Quantification of Malaria Parasites on Stained Thick and Thin Blood Films in Research Settings. Geneva, Switzerland: World Health Organization, 33.

[44] O’Connors CT Soepanto A, 1979 Kunci Kunci Bergambar Untuk Anopheles Betina Dari Indonesia. Translated and revised by Atmosoedjono S, Bangs, MJ, 1989. Illustrated key to the *Anopheles* of Indonesia. 1989 Jakarta, Indonesia: The Ministry of Health, 1–40.

[45] Detinova TS, 1962 Age-grouping methods in Diptera of medical importance with special reference to some vectors of malaria. Monogr Ser World Health Organ 47: 13–191. 13885800

[46] Lobo NF 2015 Unexpected diversity of *Anopheles* species in eastern Zambia: implications for evaluating vector behavior and interventions using molecular tools. Sci Rep 5: 17952. 26648001 10.1038/srep17952 PMC4673690

[47] St Laurent B 2016 Behaviour and molecular identification of *Anopheles* malaria vectors in jayapura district, Papua province, Indonesia. Malar J 15: 192. 27060058 10.1186/s12936-016-1234-5 PMC4826537

[48] Echeverry DF 2017 Fast and robust single PCR for *Plasmodium* sporozoite detection in mosquitoes using the cytochrome oxidase I gene. Malar J 16: 230. 28569159 10.1186/s12936-017-1881-1 PMC5452387

[49] Beier JC, 2002 Vector incrimination and entomological inoculation rates. Methods Mol Med 72: 3–11. 12125127 10.1385/1-59259-271-6:01

[50] WHO, 2016 Test Procedures for Insecticide Resistance Monitoring in Malaria Vector Mosquitoes, 2nd edition Geneva, Switzerland: World Health Organization.

[51] CDC, 2010 Guideline for Evaluating Insecticide Resistance in Vectors Using the CDC Bottle Bioassay. Atlanta, GA: The Centers.

[52] Spruance SL Reid JE Grace M Samore M, 2004 Hazard ratio in clinical trials. Antimicrob Agents Chemother 48: 2787–2792. 15273082 10.1128/AAC.48.8.2787-2792.2004 PMC478551

[53] Hernán MA, 2010 The hazards of hazard ratios. Epidemiology 21: 13–15. 20010207 10.1097/EDE.0b013e3181c1ea43 PMC3653612

[54] Alker AP 2007 *Pfmdr1* and in vivo resistance to artesunate-mefloquine in *falciparum* malaria on the Cambodian-Thai border. Am J Trop Med Hyg 76: 641–647. 17426163

[55] Ratcliff A Siswantoro H Kenangalem E Maristela R Wuwung RM Laihad F Ebsworth EP Anstey NM Tjitra E Price RN, 2007 Two fixed-dose artemisinin combinations for drug-resistant *falciparum* and *vivax* malaria in Papua, Indonesia: an open-label randomised comparison. Lancet 369: 757–765. 17336652 10.1016/S0140-6736(07)60160-3 PMC2532500

[56] Kalbfleisch JD Prentice RL, 2011 The Statistical Analysis of Failure Time Data. Hoboken, NJ: John Wiley & Sons, 462.

[57] Allison PD, 1982 Discrete-time methods for the analysis of event histories. Sociological Methodol 13: 61–98.

[58] Collett D, 2002 Modelling Binary Data. London, United Kingdom: Chapman and Hall/CRC, 408.

[59] Redmond CK Colton T, eds 2001 Biostatistics in Clinical Trials. Hoboken, NJ: John Wiley & Sons.

[60] Baird JK Bangs MJ Maguire JD Barcus MJ, 2002 Epidemiological measures of risk of malaria. Methods Mol Med 72: 13–22. 12125108 10.1385/1-59259-271-6:13

[61] World Health Organization, 2018 World Malaria Report 2018. Geneva, Switzerland: WHO.

[62] Brown CH Ten Have TR Jo B Dagne G Wyman PA Muthén B Gibbons RD, 2009 Adaptive designs for randomized trials in public health. Annu Rev Public Health 30: 1–25. 19296774 10.1146/annurev.publhealth.031308.100223 PMC2778326

[63] Chow SC Chang M, 2011 Adaptive Design Methods in Clinical Trials. London, United Kingdom: Chapman and Hall/CRC Biostatistics Series, 375.

[64] Guyatt GH Oxman AD Vist GE Kunz R Falck-Ytter Y Alonso-Coello P Schünemann HJ, 2008 GRADE: an emerging consensus on rating quality of evidence and strength of recommendations. BMJ 336: 924–926. 18436948 10.1136/bmj.39489.470347.AD PMC2335261

[65] Frieden TR, 2017 Evidence for health decision making–beyond randomized, controlled trials. N Engl J Med 377: 465–475. 28767357 10.1056/NEJMra1614394

[66] Wilson AL Boelaert M Kleinschmidt I Pinder M Scott TW Tusting LS Lindsay SW, 2015 Evidence-based vector control? Improving the quality of vector control trials. Trends Parasitol 31: 380–390. 25999026 10.1016/j.pt.2015.04.015

[67] WHO-Global Health Observatory Data Repository, 2015 Crude Birth and Death Rate Data by Country. Available at: http://apps.who.int/gho/data/view.main.CBDR2040. Accessed July 8, 2019.

[68] VCAG, Vector Control Advisory Group Available at: http://www.who.int/vector-control/vcag/en/. Accessed July 10, 2019.

[69] Lengeler C, 1998 Insecticide-treated bed nets and curtains for preventing malaria. Cochrane Database Syst Rev 2: CD000363. 10.1002/14651858.CD000363.pub2 15106149

[70] Unitaid Innovative Repellents for Disease-Carrying Mosquitoes. Available at: https://unitaid.org/project/innovative-repellents-for-disease-carrying-mosquitoes/. Accessed July 10, 2019.

