# Erratum

**DOI:** 10.4269/ajtmh.19-0554err

**Published:** 2020-10-16

In “Efficacy of a Spatial Repellent for Control of Malaria in Indonesia: A Cluster-Randomized Controlled Trial” by Syafruddin et al. (https://www.ajtmh.org/content/journals/10.4269/ajtmh.19-0554), there is an error in Figure 6. Although the caption indicates that the figure shows the mean (+SD) cumulative biweekly indoor (A) and outdoor (B) anopheline human-landing catch averaged over 20–24 households per treatment arm, the figure itself shows the indoor anopheline landing rate in both panels. A corrected Figure 6 is shown below.

**Figure 6.**
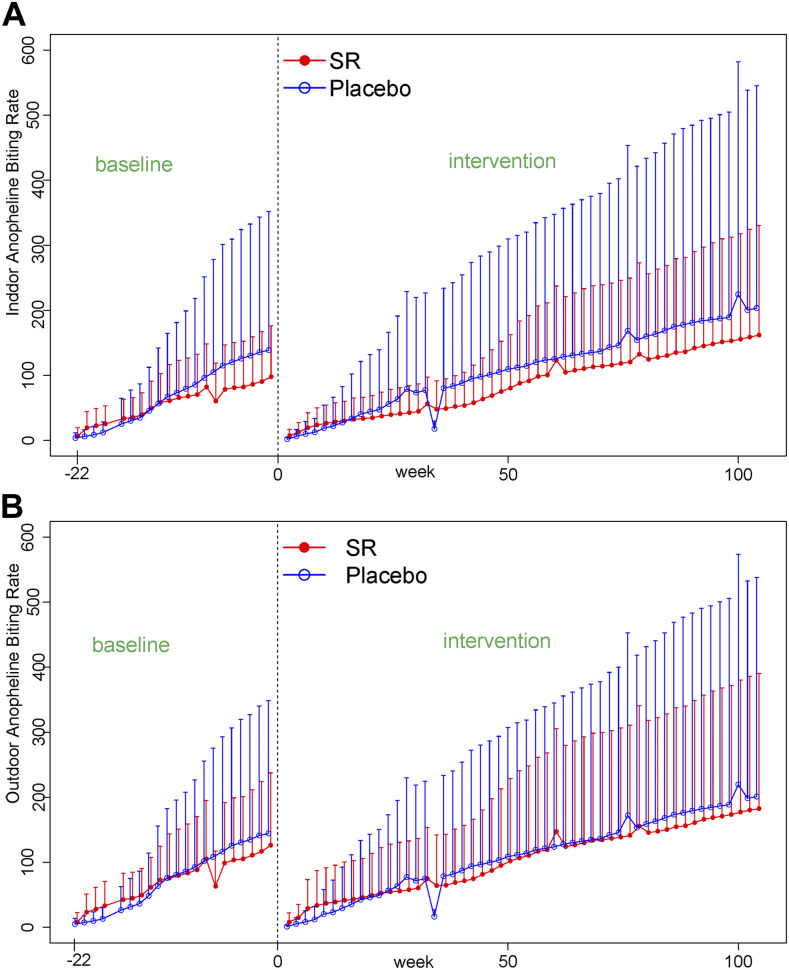
Mean (+SD) cumulative biweekly indoor (**A**) and outdoor (**B**) anopheline human-landing catch averaged over 20–24 households per treatment arm—spatial repellent intervention and placebo, respectively.

The *Journal* regrets the error.

