# Supplementary material for: Efficacy of a Spatial Repellent for Control of Malaria in Indonesia: A Cluster-Randomized Controlled Trial
Source: Am J Trop Med Hyg. 2020 May 18;103(1):344–58. doi: 10.4269/ajtmh.19-0554 (PMC7356406; doi:10.4269/ajtmh.19-0554)
Supplement: Supplementary file 1 [file tpmd190554.SD1.pdf]

Supplemental Information 1. Statistical Analyses Plan

Spatial Repellent Products for Control of Vector Borne Diseases

Statistical Analysis Plan

Version 5.1

Feb 7, 2019

Prepared by Fang Liu (internal study statistician)

fang.liu.131@nd.edu

Contents

Summary of changes in the SAP throughout its life cycle 3
1 Objectives 4
2 Hypotheses 4
3 Endpoints 4
4 Study Design 5
5 Population for analysis 5
6 Definition of new malaria infection 6
7 Statistical Methods 6
7.1 Primary endpoint (ITT Population) 6
7.2 Secondary endpoints (ITT Population) 7
7.3 Handling of missing data 10
7.4 Interim analysis 10
7.5 Analysis of baseline data 10
8 Software 10
9 Sample Size Determination 10
Reference 11
Appendix 12
I. Mock Tables and Figures 12
II. Some sample SAS and R procedures used in the analysis 15

Summary of changes in the SAP throughout its life cycle

Version	Date	Summary of Changes
V1.0	March 13, 2015	NA
V2.0	Oct 15, 2015	Added more details on the statistical methods.
V3.0	March 5, 2016	The SAP underwent a major revision as the study design changed from a group sequential design to a conventional study with one final look, and downsized from a 3-site study to one site.
V4.0	Jan 30, 2017	Addressed the comments from the DSMB statistician; removed the community effect component from the study.
V4.1	Jan 10, 2018	Added a more detailed explanation on what the sample size requirement (54 households per cluster) was based on. The sample size did not change; only that more details were provided.
V5.0	Sep 8, 2018	Addressed the comments from study PIs on the SAP, and the statistician from the DSMB statistician.
V5.1 (this version)	Feb 7, 2019	Correction of typos before finalization.

1 Objectives

Primary Objective

To evaluate the protective efficacy (PE) of spatial repellent (SR) against first-time malaria infection.

Secondary Objectives

1. To evaluate the protective efficacy (PE) of SR against overall (first-time and recurrent) malaria infections.
2. To evaluate the effect of SR on mosquito human contact using human biting rate (HBR) as an indicator.
3. To investigate the relationships between the reduction in first-time and overall malaria infection and mosquito HBR.

Tertiary Objectives

1. To evaluate the effect of SR on mosquito survival and population age structure using parity rate as an indicator.
2. To evaluate the effect of SR on mosquito infectivity using sporozoite positivity rate as an indicator.
3. To investigate the relationships between the reduction in first-time and overall malaria infection and mosquito parity rate and sporozoite positivity rate.
4. To investigate the effect of SR on HBR, parity rate, and sporozoite positivity rate by mosquito species (anopheline and culicine).
5. To evaluate the safety of the SR product in human subjects.

2 Hypotheses

Primary Hypothesis

H_0: SR does not reduce the first-time malaria hazard rate compared to placebo (PBO).

H_1: SR reduces the first-time malaria hazard rate compared to PBO (first-time malaria hazard ratio between SR and placebo is < 1; expected hazard ratio is 70% or expected PE is 30%).

Secondary Hypothesis

Estimation:

1. The hazard ratio of SR versus PBO on overall malaria infections will be estimated.
2. The change in the mosquito HBR in SR relative to PBO will be quantified.
3. The relationship between the reduction in first-time and overall malaria hazard rates and the decrease in mosquito HBR will be quantified.

3 Endpoints

- The primary endpoint is the time to first-time malaria infection collected from the follow-up period post randomization with intervention.
- The second endpoints include:
    - Overall malaria infections during intervention follow-up period.
    - Entomological endpoint: mosquito HBR.
- The tertiary endpoints include:
    - Other entomological endpoints including mosquito parity rate and sporozoite positivity rate.

- First-time and overall malaria infections diagnosed by microscopy during intervention follow-up period.
- Safety measures (the frequency of adverse events/AEs and serious adverse events/SAEs) during baseline and intervention follow-up periods.

4 Study Design

The study design is a cluster-randomized, double-blind, placebo-controlled clinical trial with 12 clusters per intervention arm (SR and PBO). Fifty-four households are recruited within each cluster (factoring in a 20% loss-to-follow-up rate during intervention). At least one child aged from <6 to 59 months old from each household is recruited for active screening every 4 weeks for malaria infection during the follow-up period.

5 Population for analysis

The intention to treat (ITT) analysis is the primary analysis approach for both the primary and secondary objectives. The ITT population includes the first recruited kid from each recruited household that has at least one blood sample post randomization. If a recruited subject comes from a household used for entomological data collection, that subject will be not used in the ITT analysis. The per-protocol (PP) analysis is included as a supplementary analysis for the primary and secondary objectives. The PP population includes the subjects from the ITT population that are treated following the specifications of the study protocol without major protocol deviations. In addition, the following rules are applied to deal with the subjects who experience events listed as such.

5.1 Subjects who moved to a new house during the intervention follow-up period and not used as replacement

For a subject who moved to a different house within the same cluster, that subject will be included in both the ITT and PP analyses. The household characteristics will be updated at the time the subjects moved. For a subject who moved to a different cluster due to reasons not related to the treatment assignment, the data from the subject before moving will be included in the ITT analysis. The data before and after moving from the subject will be included in the PP analysis, but both the treatment information and the baseline household characteristics will be updated at the time the subjects moved.

5.2 Subjects who were hospitalized for serious complicated illness (e.g. chronic illness), died, dropped out, or missed scheduled visits due to reasons not related to the malaria outcome or intervention during the follow-up period

For subjects that fall under this category, the available data from the subjects (up to the time point when the subjects were hospitalized, died, or dropped out; data from the scheduled visits that the subjects did not miss) before the loss-to-follow-up will be included in both the ITT and PP analyses (see Section 6.4 of the SAP for more details).

5.3 Subjects who did not receive (complete) intervention due to travelling outside, mis-application or partial application of the product

For the ITT analyses, these subjects will be included as is. For the supplementary PP analysis, “travel outside” (Y or N; an individual-level covariate) and the product application rate in each household (expected to be close to 100%) will be included as covariates if the data are not overly imbalanced between the Y and N categories for “travel outside”, and there is practically/clinically meaningful variation in the product application rate across households and clusters.

5.4 Replacement subjects

Replacement subjects are defined as subjects who were recruited into the study and cleared of parasites with radical cure at a time point after intervention to replace initially recruited loss to follow

up (LTFU) subjects to maintain minimum cohort numbers. If the replacement occurs in the baseline period or before the first scheduled visit of the subjects who they replace, then the data from the replacement subjects will be included in the primary analysis. Data from replacement subjects will not be included in the primary analysis for PE if the replacement of the original subject (from the same cluster) occurred after the original subject he/she replaced has least one blood sample after randomization. However, a supplementary analysis will be performed that includes the replacement subjects.

6 Definition of new malaria infection

Following a true positive malaria screening, whether the next positive malaria infection, either during active or passive screening, is a new infection or just a remnant or a carryover from the previous malaria infection depends on the time lapse between the two malaria infections, and whether and when the first malaria is treated.

- If the second malaria infection occurs beyond 3 weeks from the treatment date of the first malaria, then the second malaria infection will be regarded as a new infection; otherwise, it will not be regarded as a new infection.
- If there is no treatment for the first infection, and the second malaria infection occurs beyond 3 weeks from the first malaria infection with at least negative active screening between the two positives, then it will be regarded as a new infection; otherwise, it will not be regarded as a new infection.

The positive diagnosis that cannot be treated as a new infection will be re-coded as negative before any the following statistical analysis on malaria infection (baseline, first-time infection, overall malaria infection) is applied.

7 Statistical Methods

7.1 Primary endpoint (ITT Population)

The primary hypothesis on PE against first-time malaria infection will be tested by comparing the hazard rates of the first-time malaria infection between SR and PBO upon the completion of the study in the ITT population. The complementary log-log (cloglog) regression model $\log\left(-\log\left(1-\theta_{kjit}\right)\right)=\beta_{0t}+\boldsymbol{x}_{kjit}^{t}\beta+z_k$ will be applied [1-4]. θ_{kjit} is the discrete time hazard rate of subject i from household j in cluster k at time t, and $\boldsymbol{x}_{kjit}$ contains visit (as a categorical predictor), the individual-level (age, gender), household-level (number of doors, open eaves Y or N, wall type), and cluster-level (baseline incidence rate, cluster population size, intervention group) covariates. First-order interactions terms will also be included in the model if deemed scientifically or statistically relevant. If the data are extremely unbalanced in a categorical covariate (e.g., 99% households had the same type of walls) or if a non-ignorable portion of the subjects have missing values on a covariate (due to missing at random/MAR or missing completely at random/MCAR), that covariate may be excluded in the model. $z_k \sim N(0,\sigma_1^2)$ is the random effect at the cluster level respectively.

PE is estimated by $(1-\exp(\hat{\beta}))\times 100\%$, where $\hat{\beta}$ is the estimated regression coefficient associated with the treatment group, and $\exp(\hat{\beta})$ is the estimated hazard ratio (HR) between SR and placebo, with a 90% confidence interval (CI) based on the Wald test (90% CI is obtained instead of 95% CI as the primary hypothesis is one-sided). The cloglog model is a proportional hazard model and thus HR does not depend on time t. The null hypothesis of PE = 0% is equivalent to $\beta=0$, which will be tested by the Wald's test $z=\hat{\beta}/s$, where s is the estimated standard error of $\hat{\beta}$. It will be concluded that SR reduces the first time malaria hazard rate compared to placebo

if $z < z_{0.05} = -1.645$ (as the primary hypothesis is one-sided); otherwise, the study does not have enough evidence to suggest that SR reduces the first time malaria hazard rate compared to placebo at a one-sided significance level of 5%.

The Kaplan-Meier (KM) curves on the first-time malaria infection per cluster will be provided for the SR and PBO arms respectively.

Also noted is that the active screening of malaria occurs every 4 weeks and the passive screening occurs in between two active screenings only when a subject experiences a fever. It is possible there are only a few passive screenings upon the completion of data, leading to data imbalance between the odd-numbered visits (the active screening) and the even-numbered visits (the passive screening). To deal with this problem if it occurs, we will apply the following approach. If the passive screening in a visit is negative on malaria, then that data point will be removed as it contains no additional info on malaria or time at risk on top of the active screenings before and after it. If the passive screening is positive, then the passive positive will be assigned to either the active screening visit immediately before the passive screening or after, whichever is closer to the passive screening in time. The same approach will be applied to the analysis of overall malaria infections in Sec 7.2.

7.2 Secondary endpoints (ITT Population)

PE of SR protection against the overall malaria infections

The second endpoint on PE of SR protection against the overall malaria infections will be estimated by comparing the hazard rates of overall malaria infections between SR and PBO upon the completion of the study in the ITT population. The cloglog regression model $\log(-\log(1-\theta_{kjit})) = \beta_{0t} + \boldsymbol{x}_{kjit}^{t}\beta + z_k + z_{j(k)}$ will be applied [1-4]. θ_{kjit} is the discrete time hazard rate of subject i from household j in cluster k at visit t, and $\boldsymbol{x}_{kjit}$ contains visit, the individual-level (age, gender), household-level (number of doors, open eaves Y or N, wall type), and cluster-level (baseline incidence rate, cluster population size, intervention group) covariates. First-order interactions terms will also be included in the model if deemed scientifically or statistically relevant. If the data are extremely unbalanced in a categorical covariate (e.g., 99% households had the same type of walls vs 1% that didn't) or if a non-ignorable portion of the subjects have missing values on a covariate (due to MAR or MCAR), that covariate may be excluded in the model. $z_k \sim N(0, \sigma_1^2)$ and $z_{j(k)} \sim N(0, \sigma_2^2)$ are the random effects at the cluster and individual levels respectively. PE against the overall malaria infections is estimated by $(1-\exp(\hat{\beta})) \times 100\%$, where $\hat{\beta}$ is the estimated regression coefficient associated with the treatment group, and $\exp(\hat{\beta})$ is the estimated hazard ratio (HR) between SR and placebo, with a 90% CI based on the Wald test.

Entomological effects of SR

The entomological data are collected in a subset of 12 randomly selected clusters. Clusters will be stratified by treatment arm to ensure balanced recruitment (6 clusters in each treatment group with 4 households per cluster). The endpoints in the entomological analysis include daily mosquito HBR (number of mosquitos caught during the 12-hr interval overnight), daily parity rate and daily sporozoite positivity rate (among the mosquitos caught during the 12-hr interval overnight), measured every 2 weeks. The time profile plots of each of entomological endpoints will be obtained over the intervention follow-up period.

An appropriate statistical model will be identified after examining the distributional characteristics of the HBR data, which is likely to be a (zero-inflated) Poisson distribution or a (zero-inflated) negative binomial distribution if there is over-dispersion. The covariates in the model for analyzing

HBR will include the fixed effects of intervention group, the interaction between treatment and location of collection (inside or outside), visit (as categorical), baseline incidence rate, baseline vector count, cluster population, and random effects for household nested within cluster and for cluster. Statistically significant and relevant interaction terms will also be included in the models. The ratio between SR and placebo in HBR will be estimated, and the %change in HBR by SR is given by (1-HBR ratio)×100%.

The model for parity rate will be the (zero-inflated) Poisson distribution or a (zero-inflated) negative binomial distribution with the daily porous mosquitos s as the outcome and the daily HBR as the offset, and the same set of covariates as those used in the model for analyzing HBR. The model for the sporozoite positivity rate will be similar to the parity rate with the change of outcome variable to daily mosquitos with positive sporozoite. Note that if the data on parity and sporozoite positivity are highly unbalanced (e.g., 99% nulliparous or 99% negative sporozoite), then the model might lead to unstable estimates or the model might not even converge. In such cases, only summary statistics will be provided.

Relationship between epidemiological and entomological endpoints for anopheline mosquitos

To explore the relationship between the epidemiological and the entomological endpoints for putative malaria mosquitos, a similar model as the cloglog models used to address the primary hypothesis for the first-time malaria infection and the secondary endpoint for the overall malaria infection will be applied to the epidemiological and entomological data in the 12 clusters from which the entomological data are collected. Besides the covariates and random effects specified in each case, the model will also include a variable that captures the entomological information. For HBR, the measurement to be paired with a malaria diagnosis in an individual is average daily HBR taken within 7 to 28 days before the diagnosis over the two-week period and over the 4 sentinel households in the same cluster to which the individual belongs to. The regression coefficient associated with the covariate log(HBR) quantifies the change in the hazard rate on the log scale, given one unit increase in log(HBR). In addition, a similar model but with the interaction between treatment and log(HBR) will also in run. The regression coefficient associated with the interaction term approximately quality the change in PE with one unit increase in log(HRB) (that is, how HBR affects the PE). For parity rate and sporozoite positivity rate, as long as there is enough data collected on these two endpoints and they not highly unbalanced (e.g., 99% mosquitos caught are nulliparous or sporozoite negative), the relationship between the malaria hazard rate and those two will also investigated in a similar fashion as for HBR. Supplementary analysis

Per-Protocol population

The primary and secondary analyses laid out in Sections 6.1 and 6.2 for the first-time infection, the overall infections, and the examination of relationship between the ento- and epi- endpoints will also be carried out in the PP population with some modification on the covariate list in the cloglog models. Specifically, for the PP analysis, "bednet usage" in the last 24 hrs (Y or N), "travel outside" (Y or N; an individual-level covariate), and the product application rate in each household (expected to be close to 100%) will be included as covariates if the data are balanced between the Y and N categories on these two covariate, and there is practically/clinically meaningful variation in the product application rate across households and clusters.

A supplementary analysis on the PE against the first-time infection and overall infections will be performed by including the replacement subjects in the model if the replacement occurs after the first scheduled visit of the original subjects who they replace. If replacement subjects have been under intervention exposure, the analysis will assume the previous exposure does not affect the

time to the first-time infection. If replacement subjects come from the same households as the original subjects, the random effects $z_{j(k)} \sim N(0, \sigma_2^2)$ that account for the within-household correlation will be included.

Incidence rate

The first-time and overall malaria incidence rates per person-year during the whole intervention follow-up will be calculated by cluster for the SR and the PBO arms respectively. The first-time malaria incidence rate is defined as the ratio of the number of first-time malaria cases during the whole study vs sum of the time to event/time at risk (in year) across the individuals within the same cluster, and the overall malaria incidence rate is defined as the ratio of the number of new malaria cases during the whole study vs sum of the time to event/time at risk (in year) for each of the new cases across the individuals within the same cluster.

Since the active screenings of malaria incidences are either every 4 weeks (active screening) with passive screening taken between two active screenings if fever is reported, the actual time for contracting malaria is unknown (interval censored). Therefore, the mid-point between two consecutive screenings will be used as the time at risk for a malaria event that occurs in the latter screening. The average per-person-year first-time and overall malaria incidence rates in the SR and the PBO arms, and the incidence ratios between the two will be calculated, together with the coefficients of variation in both arms on both incidence endpoints.

Analysis of the relationship between incidence rate and entomological endpoints

We will also examine the relationship between first-time and overall malaria incidence rates per person-year with the entomological endpoints. Toward that end, we will aggregate the incidence rate as well as the entomological endpoints every 4 week. Specifically, the first-time infection incidence rate will be calculated as the number of first-time infections every 4 week divided by the sum of time at risk across all the subjects for first-time infection during that period by cluster, and the overall infection incidence rate will be calculated as the number of new infections every 4 week divided by the sum of time at risk across the subjects or new infections during that period by cluster. The HBR to be matched with the incidence rates will a weighted average of the HBR collected over a 6-week period as depicted in the table below.

Week		-2	-1	0	1	2	3	4
malaria incidence								
weights of the HBR data to be matched with incidence rates	Wk 4 incidence				1/3	1/3	1/3	
	Wk 3 incidence			1/3	1/3	1/3		
	Wk 2 incidence		1/3	1/3	1/3			
	Wk 1 incidence	1/3	1/3	1/3				
	**overall**	**1/3**	**2/3**	**1**	**1**	**2/3**	**1/3**	

Scatter plots of the first-time and new malaria incidence rates versus HBR will be plotted. Appropriate models might also be adopted to examine the relationship between HBR and malaria incidence rates. Similar analysis will be applied to analyze the relationship between incidence rate vs parity rate, and vs sporozoite positivity rate, respectively, if sufficient data on parity and sporozoite positivity are collected.

VCAG's review comments

Per VCAG's review comments, a supplementary analysis on the first-time infection and the overall infections will be also performed by removing all the baseline covariate from the cloglog models presented Sec 6.1 and 6.2 and only keeping "intervention group" as the only covariate in addition

to visit (as a categorical predictor). The hazard ratios between SR and PBO will be provided, along with 90% CIs.

<u>AEs and SAEs</u>

AEs and SAEs will be tabulated and documented.

7.3 Handling of missing data

Per protocol, the subjects are screened actively on their malaria status (the outcome) every four weeks.

- If a subject missed one or more scheduled visits, the subject will have missing values on the outcome that can be regarded as ignorable missingness.
- If a subject drops out study due to reasons unrelated to the SR product and/or malaria infection, then the missing observations from the subject can be regarded as ignorable missingness.

In both cases, all available data from the subject will be included in the primary and secondary analysis, without employing any specific technique to deal with the data.

If a non-ignorable portion of the subjects have missing values on a covariate (due to missing at random or missing completely at random), that covariate maybe may be excluded in the model.

7.4 Interim analysis

No formal interim analysis will be performed in this study.

7.5 Analysis of baseline data

The per-person-year first-time and overall malaria incidence rates from the 24 recruited clusters will be calculated. Since the malaria incidences are collected periodically, the mid-point between two visits will be imputed as the time at risk for a new malaria infection. The average incidence rate across clusters will be calculated, together with the coefficient of variation.

8 Software

Software used will be SAS for Windows, Version 9.4 or higher (SAS Institute, Cary, NC, USA) and Rstuduo Version 1.0.143 or higher (RStudio, Inc, Boston, MA, USA).

9 Sample Size Determination

The sample size determination on the required number of households per cluster is based on the hazard rate comparison in the proportional hazards regression model [20, 21]. The required number of events is 417 to reach 80% power in testing the primary hypothesis, *assuming a 1-sided type I error rate = 5%, true PE = 30%, and between-cluster coefficient of variance (CV) of baseline hazard rate = 25%* (corresponding to a design effect of ~2.15). With 12 clusters per treatment, 2-month accrual with 22 months follow/up (a 2-year study), and an assumed baseline hazard rate of 0.3 per person year, then the required sample size is 45 households per cluster. Factoring in a 20% loss to follow-up rate, the total sample size required in terms of the number of households is 54 households per cluster.

Reference

1. J. D. Kalbfleisch, R. L. Prentice (2002, 2nd ed), The Statistical Analysis of Failure Time Data, Wiley-Interscience
2. P. D. Allison (1982), Discrete-Time Methods for the Analysis of Event Histories, Sociological Methodology, 13: 61-98
3. D. Collett (2002, 2nd ed), Modelling Binary Data, Second Edition, Chapman & Hall
4. Carol K. Redmond & Theodore Colton (2001; 1st Edition), Biostatistics in Clinical Trials, Wiley
5. C. Law and R. Brookmeyer (1992). Effects of mid-point imputation on the analysis of doubly censored data. Statistics in Medicine, 11:1569-1578.
6. B. P. Carlin and J. S. Hodges (1999), Hierarchical proportional hazards regression models for highly stratified data, Biometrics.55(4):1162-1170
7. W. Pan (2000), A Multiple Imputation Approach to Cox Regression with Interval-Censored Data. Biometrics, 56(1): 199-203
8. V. Henschell, J. Engel, D. Hölzel and U. Mansmannina (2009), A semiparametric Bayesian proportional hazards model for interval censored data with frailty effects, BMC Medical Research Methodology, 9:9
9. S. Lang (2004), Brezger A: Bayesian P-Splines. Journal of Computational and Graphical Statistics, 13:183-212.
10. G. Yin and J. G. Ibrahim (2005), A Class of Bayesian Shared Gamma Frailty Models with Multivariate Failure Time Data, Biometrics, 61(1):208-216
11. R. J .A. Little and D. B. Rubin (2002), Statistical Analysis with Missing Data (2nd edition), J. Wiley & Sons, New York.
12. D. B. Rubin (1976), Inference and missing data. Biometrika, 63, 581-592.
13. D. B. Rubin (1987), Multiple Imputation for Nonresponse in Surveys. J. Wiley & Sons
14. G. Berry (1993), The analysis of mortality by the subject-years method, Biometrics, 39:173-184
15. E. J. Atkinson, C. S. Crowson, R. A. Pedersen, T. M. Therneau (2008), Poisson models for person-years and expected rates, technical report #81, Mayo foundation.
16. C. E. McCulloch and J. M. Neuhaus (2005). Generalized Linear Mixed Models in Encyclopedia of Biostatistics, John Wiley and Sons,
17. A. Agresti (2013). Categorical Data Analysis, 3rd ed. John Wiley & Sons.
18. N. E. Breslow and D. G. Clayton (1993). Approximate inference in generalized linear mixed models. Journal of the American Statistical Association, 88(421), pp 9-25,
19. A. Skrondal and S. Rabe-Hesketh (2004). Generalized Latent Variable Modeling: Multilevel, longitudinal, and structural equation models. Chapman & Hall/CRC Press.
20. M. Lievens, J. J. Aponte, J. Williamson, B. Mmbando, A. Mohamed, P. Bejon and A. Leach (2011), Statistical methodology for the evaluation of vaccine efficacy in a phase III multi-centre trial of the RTS,S/AS01 malaria vaccine in African children, Malaria Journal, 10:222
21. The RTS,S Clinical Trials Partnership (2011), First Results of Phase 3 Trial of RTS,S/AS01 Malaria Vaccine in African Children, The New England Journal of Medicine, 365(20): 1863-1875

Appendix

I. Mock Tables and Figures

Figure 1: flow diagram of progress of clusters and individuals (From Campbell (2010): *Consort 2010 statement: extension to cluster randomized trials*)

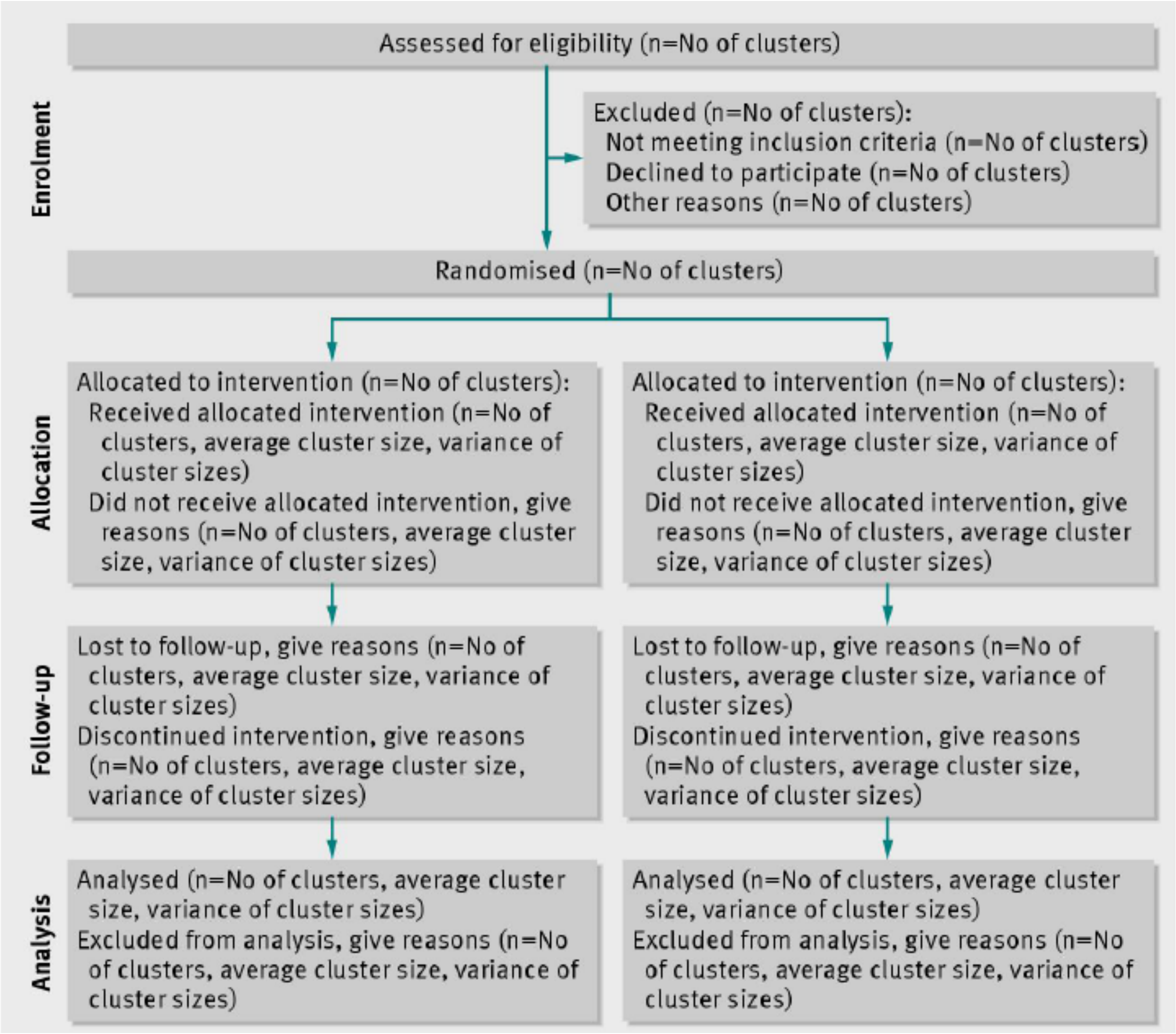

Table 1: Summary on baseline covariates

	SR	Placebo
Individual level		
age (mean ± SD, n)<br>gender (% of boys, n)		
household level		
Baseline spray (Yes%, n)<br>house wall type (%, n)<br>house roof type (%, n)<br>open eaves (Yes%, n)<br>floor height (mean ± SD, n)<br># of windows (mean± SD, n)<br># of doors (mean± SD, n)		
Cluster level		
Cluster size (mean± SD, n)<br>Baseline incidence rate (mean± SD, n)		

Table 2: Protective Efficacy (PE) of SR against 1st-time infection

Treatment	Baseline incidence rate	# of households	# of events#	hazard ratio (95% CI)	PE (95% CI)
SR					
placebo					

1st-time infection

Baseline coefficient of variation (CV) of incidence ate: xxx%

A similar table will be provided for overall infections

Table 3: Effects of SR compared to blank on the HBR, parity rate, sporozoite positivity rate

Endpoint	Mean (95% CI)		Ratio (95% CI)
	SR	Blank	SR vs. blank
HBR<br>Parity rate<br>sporozoite positivity rate			Only if model-based analysis is performed

Figure 2: Kaplan Meier Curves for SR and PBO on 1st-time malaria infections

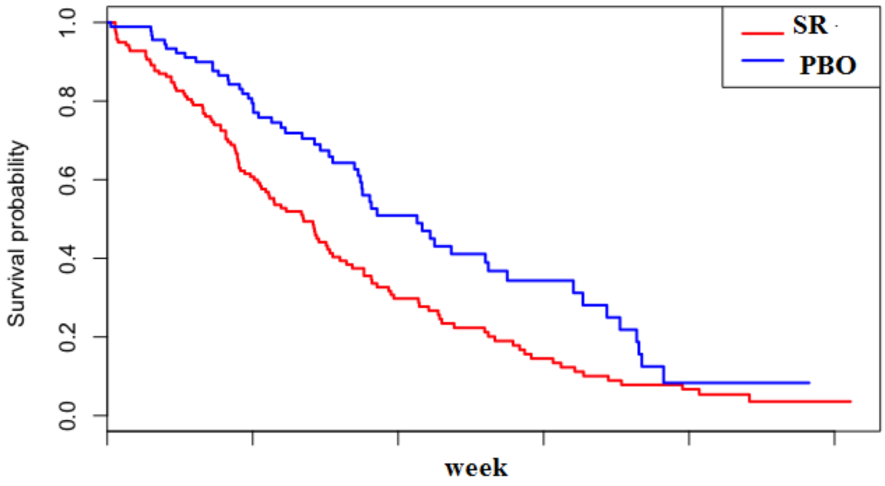

Figure 3: time profile of estimated HBR (time unit is every 2 weeks)

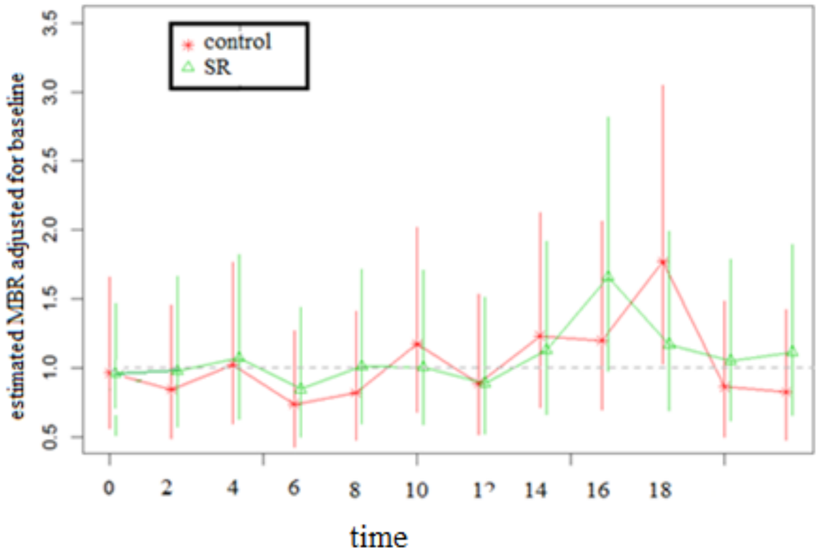

II. Some sample SAS and R procedures used in the analysis

Note the final codes for KM curves and estimation if PE could differ slightly from the sample codes below, which are meant to demonstrate the main procedures/commands in R and SAS to run the those two types of analyses rather than to be followed strictly.

a) KM curves for each cluster. Some sample codes are given below.

```
library(interval)
fit<-icfit(Surv(left,right,type="interval2")~treatment, data=malaria)
plot(fit)
```

b) For estimating the PE of SR against first-time and overall malaria infection SAS procedure `PROC glimmix` with the `cloglog` link. Each subject will have multiple rows, one for each visit. The statement `random` will be included to take account of the dependency of the subjects from the same cluster. Some sample codes are given below.

```
proc glimmix data=malaria;
   class gender visit Local_Subject_ID treatment open_eave wall_type
   cluster;
   model malaria (event='1')= visit age_first_follow_up gender
   number_doors open_eave wall_type treatment baseline_incidence
   cluster_size /dist=binary solution link=cloglog;
   random int / subject=cluster;
   estimate 'treatment' treatment 1 -1 /alpha=0.1 cl exp;
   ods output estimates=treatdiff;
run;
```
