# Supplementary material for: Efficacy of a Spatial Repellent for Control of Malaria in Indonesia: A Cluster-Randomized Controlled Trial
Source: Am J Trop Med Hyg. 2020 May 18;103(1):344–58. doi: 10.4269/ajtmh.19-0554 (PMC7356406; doi:10.4269/ajtmh.19-0554)

Supplemental Information 2. Usage of long-lasting insecticidal nets (LLIN) during baseline and intervention follow-up periods of the trial, in both the spatial repellent (SR) and placebo arms.

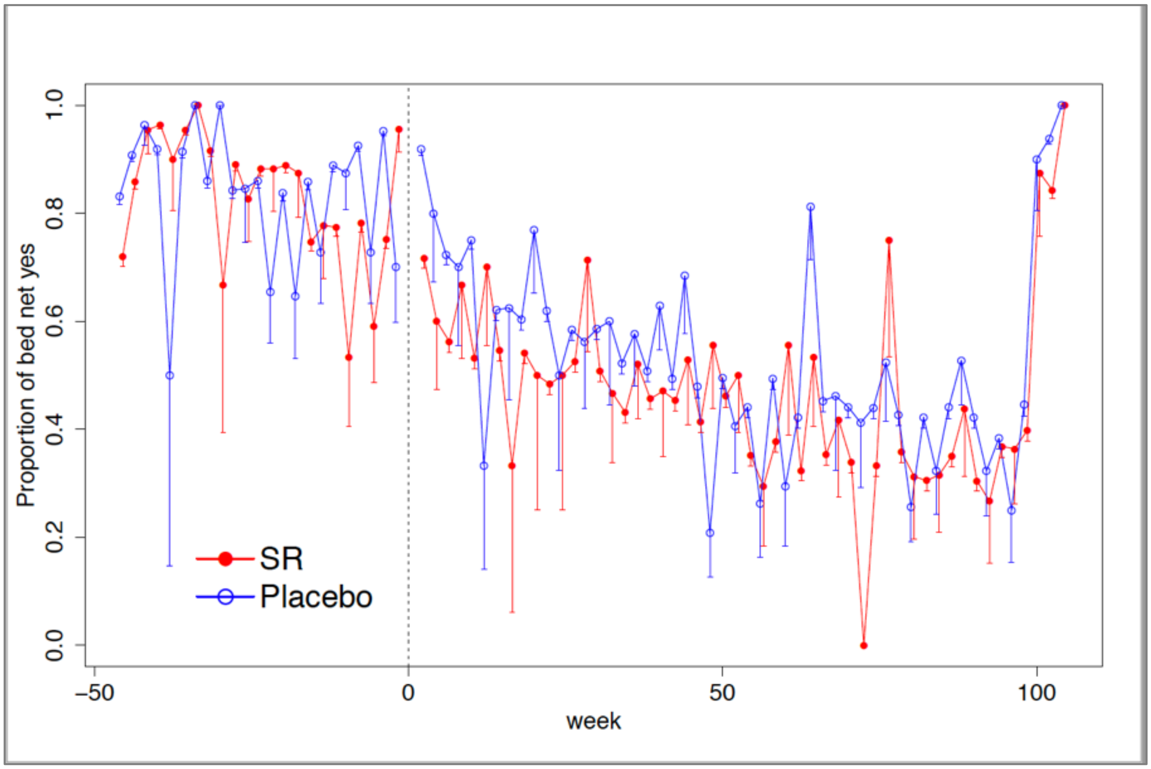

Supplement: Supplementary file 2 [file tpmd190554.SD2.pdf]
