# Supplementary material for: Efficacy of a Spatial Repellent for Control of Malaria in Indonesia: A Cluster-Randomized Controlled Trial
Source: Am J Trop Med Hyg. 2020 May 18;103(1):344–58. doi: 10.4269/ajtmh.19-0554 (PMC7356406; doi:10.4269/ajtmh.19-0554)
Supplement: Supplementary file 3 [file tpmd190554.SD3.pdf]

Supplemental Information 3. Frequency (percentage) of anopheline species captured during sentinel human-landing catch (HLC) based on adult morphological character identification*.

Anopheles species	Baseline Period				Intervention Period			
	Indoor		Outdoor		Indoor		Outdoor	
	SR	Placebo	SR	Placebo	SR	Placebo	SR	Placebo
**Total**	**2243 (100%)**	**3327 (100%)**	**2896 (100%)**	**3462 (100%)**	**3883 (100%)**	**4897 (100%)**	**4372 (100%)**	**4834 (100%)**
An. aconitus	467 (20.82%)	80 (2.40%)	768 (26.52%)	78 (2.25%)	2015 (51.89%)	173 (3.53%)	2249 (51.44%)	162 (3.35%)
An. annularis	55 (2.45%)	633 (19.03%)	70 (2.42%)	567 (16.38%)	82 (2.11%)	544 (11.11%)	99 (2.26%)	493 (10.20%)
An. balabacensis	0 (0%)	0 (0%)	0 (0%)	0 (0%)	2 (0.05%)	1 (0.02%)	0 (0%)	0 (0%)
An. barbirostris	41 (1.83%)	148 (4.45%)	49 (1.69%)	132 (3.81%)	62 (1.60%)	360 (7.35%)	56 (1.28%)	322 (6.66%)
An. barbumbrosus	0 (0%)	0 (0%)	0 (0%)	0 (0%)	1 (0.03%)	0 (0%)	0 (0%)	3 (0.06%)
An. flavirostris	355 (15.83%)	417 (12.53%)	523 (18.06%)	504 (14.56%)	757 (19.50%)	430 (8.78%)	1001 (22.90%)	482 (9.97%)
An. indefinitus	0 (0%)	1 (0.03%)	0 (0%)	1 (0.03%)	2 (0.05%)	12 (0.25%)	4 (0.09%)	20 (0.41%)
An. karwari	0 (0%)	0 (0%)	0 (0%)	0 (0%)	0 (0%)	0 (0%)	1 (0.02%)	0 (0%)
An. kochi	47 (2.10%)	501 (15.06%)	36 (1.24%)	392 (11.32%)	90 (2.32%)	803 (16.40%)	88 (4.71%)	836 (17.29%)
Leucosphyrus Group	0 (0%)	1 (0.03%)	0 (0%)	0 (0%)	2 (0.05%)	16 (1.61%)	1 (0.02%)	26 (0.54%)
An. maculatus	61 (2.72%)	88 (2.65%)	95 (3.28%)	88 (2.54%)	209 (5.38%)	79 (1.61%)	206 (4.71%)	94 (1.94%)
An. montanus	0 (0%)	0 (0%)	0 (0%)	0 (0%)	1 (0.03%)	0 (0%)	1 (0.02%)	0 (0%)
An. parangensis	0 (0%)	0 (0%)	0 (0%)	0 (0%)	0 (0%)	1 (0.02%)	0 (0%)	0 (0%)
Hyrcanus Group	0 (0%)	0 (0%)	0 (0%)	0 (0%)	0 (0%)	4 (0.08%)	0 (0%)	2 (0.04%)
An. subpictus	15 (0.67%)	7 (0.21%)	13 (0.45%)	15 (0.43%)	3 (0.08%)	105 (2.14%)	10 (0.23%)	85 (1.76%)
An. sundaicus	1001 (44.63%)	401 (12.05%)	1154 (39.85%)	562 (16.23%)	136 (3.50%)	61 (1.25%)	128 (2.93%)	62 (1.28%)
An. tessellatus	94 (4.19%)	317 (9.53%)	89 (3.07%)	295 (8.52%)	205 (5.28%)	1246 (25.44%)	192 (4.39%)	1215 (25.13%)
An. umbrosus	0 (0%)	0 (0%)	1 (0.03%)	0 (0%)	0 (0%)	0 (0%)	0 (0%)	0 (0%)
An. vagus	107 (4.77%)	733 (22.03%)	98 (3.38%)	828 (23.92%)	315 (8.11%)	1061 (21.67%)	334 (7.64%)	1031 (21.33%)
Unknown	0 (0%)	0 (0%)	0 (0%/)	0 (0%)	1 (0.03%)	1 (0.02%)	2 (0.046%)	1 (0.02%)

*Misidentification possible depending on physical condition of specimen, molecular methods required to separate out some members in species groups and complexes.
